# Herpes Zoster Vaccines

**DOI:** 10.1093/infdis/jiab387

**Published:** 2021-09-30

**Authors:** Ruth Harbecke, Jeffrey I Cohen, Michael N Oxman

**Affiliations:** 1 Department of Veterans Affairs (VA) San Diego Healthcare System, San Diego, California, USA; 2 Department of Medicine, University of California San Diego, San Diego, California, USA; 3 Laboratory of Infectious Diseases, National Institute of Allergy and Infectious Diseases, National Institutes of Health, Bethesda, Maryland, USA; 4 Department of Pathology, University of California San Diego, San Diego, California, USA

**Keywords:** herpes zoster, zoster vaccine, live attenuated Oka varicella-zoster vaccine, recombinant zoster vaccine

## Abstract

Herpes zoster (HZ) affects approximately 1 in 3 persons in their lifetime, and the risk of HZ increases with increasing age. The most common, debilitating complication of HZ is the chronic neuropathic pain of postherpetic neuralgia (PHN). Two herpes zoster vaccines, a live-attenuated varicella-zoster virus (VZV) vaccine (zoster vaccine live [ZVL]; ZOSTAVAX [Merck]) and an adjuvanted VZV glycoprotein E (gE) subunit vaccine (recombinant zoster vaccine [RZV]; SHINGRIX [GlaxoSmithKline]) are licensed for the prevention of HZ and PHN in healthy older adults. The safety and efficacy of both vaccines has been demonstrated in clinical trials in immunocompetent adults and in selected immunocompromised persons and persons with immune-mediated diseases. Numerous real-world effectiveness studies have confirmed the safety and effectiveness of both ZVL and RZV. Recombinant zoster vaccine (RZV) is more effective for prevention of HZ than ZVL. Recombinant zoster vaccine is nonreplicating and is thus safe in immunocompromised persons. Additional zoster vaccines are in different stages of development. Wider distribution of safe and effective zoster vaccines will improve the health and well being of the rapidly growing population of older adults around the world.

## Background

Varicella-zoster virus (VZV) is a neurotropic human herpesvirus that causes 2 distinct diseases, varicella (chickenpox) and herpes zoster ([HZ]; shingles). Primary VZV infection results in varicella, characterized by a generalized pruritic rash that progresses rapidly from macules to papules to vesicular lesions and then crusts. The lesions are individual and scattered, reflecting viremic spread to the skin. In healthy children, varicella is generally mild [1, 2]. In temperate climates, in the absence of varicella vaccination, varicella typically occurs in early childhood, with >90% of children infected before adolescence [2–4]. In tropical climates, primary VZV infection is delayed, commonly occurring in adolescents and adults, who experience significant morbidity and mortality [3, 5, 6]. Consequently, outbreaks of varicella have occurred among healthcare workers from tropical countries who have not had varicella [7–9].

During primary infection, VZV establishes lifelong latency in sensory and autonomic ganglia [10, 11]. Varicella-zoster virus also establishes latency in ganglia in the enteric nervous system, which may be infected by viremia or by axonal transport from neurons in sensory ganglia [12]. Recovery from varicella is associated with VZV-specific T cell-mediated immunity (VZV-CMI), which is also essential for limiting reactivation and replication of latent VZV, and thus for preventing HZ [13]. The mechanisms by which VZV establishes, maintains, and reactivates from latency are poorly understood.

**Figure 1.**
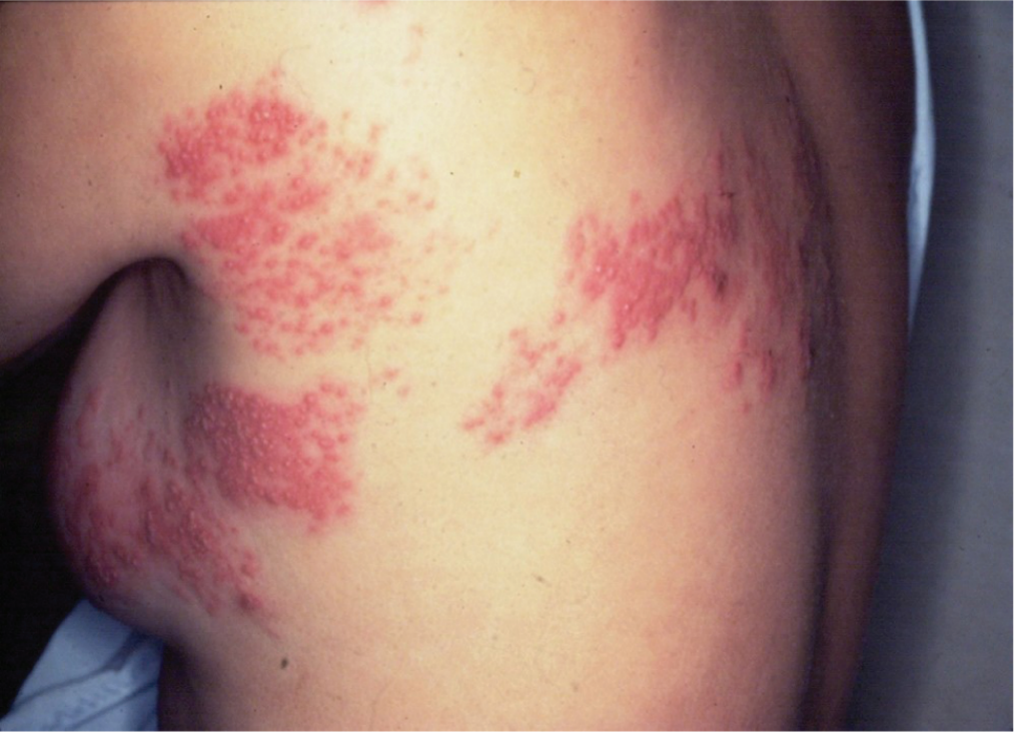
Classical herpes zoster in the left T4 dermatome. Note the concentration of lesions in areas of the dermatome innervated by the posterior primary division and the lateral branch of the anterior primary division of the left T4 spinal nerve. Source: Levin et al [2].

Reactivation from latency and subsequent replication of VZV results in HZ, a localized disease of the sensory ganglion, nerve, and skin. Herpes zoster manifests as unilateral radicular pain and a vesicular rash that is generally limited to the dermatome innervated by a single dorsal root or cranial nerve ganglion [14–17]. The lesions are clustered, reflecting intraneural transmission to the skin (Figure 1). Herpes zoster occurs sporadically throughout the year without seasonal prevalence, independent of the prevalence of varicella. There is no evidence that HZ results from exogenous VZV infection, ie, that HZ can be acquired by contact with a person with varicella or HZ. Rather, the incidence of HZ is determined by factors influencing the virus-host relationship, primarily the host’s VZV-CMI, which maintains VZV latency.

### Natural History of Herpes Zoster

**Figure 2.**
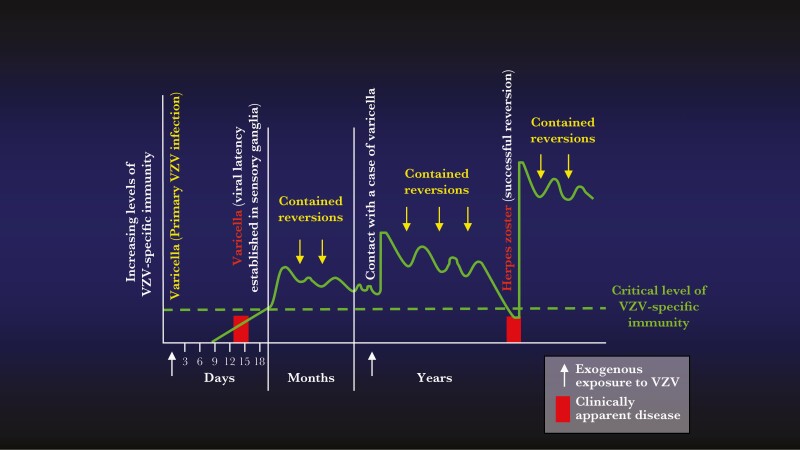
During varicella, varicella-zoster virus (VZV) establishes life-long latency in neurons in sensory ganglia. Herpes zoster results when latent virus reactivates, multiplies in the ganglion, and spreads back down the sensory nerve. Varicella induces immunity to VZV that prevents reactivating virus from multiplying, thus preventing herpes zoster. This immunity decreases over time until it falls below a critical threshold, permitting reactivating VZV to multiply and cause herpes zoster. The age-related decline in VZV-specific immunity is slowed by periodic stimulation due to exogenous exposure to VZV from contact with varicella and by episodes of reactivation of latent VZV where host immunity inhibits virus multiplication before it can cause herpes zoster (contained reversions). Modified from R Edgar Hope-Simpson [15].

Observations by R. Edgar Hope-Simpson [15], reported in a landmark publication in 1965, provided the rationale for the development of a vaccine to prevent HZ and its complications. Carefully following every case of varicella and HZ in his clinical practice for 16 years, Hope-Simpson observed that the incidence and severity of HZ and PHN increased with increasing age [15]. He hypothesized that primary infection with VZV (ie, varicella) establishes lifelong latent VZV infections in sensory neurons, and also induces immunity to VZV that limits the ability of the latent virus to reactivate and multiply to cause HZ (Figure 2). He further postulated that this immunity to VZV decreases over time until it falls below a critical threshold, permitting latent VZV to reactivate, multiply, and spread, resulting in HZ. Hope-Simpson [15] also proposed that subclinical VZV reinfection caused by exposure to varicella, as well as spontaneous ganglionic reactivations that are contained by host immunity to VZV before HZ can develop (contained reversions), both stimulate the host’s immunity to VZV, delaying its decline to levels permitting latent VZV to reactivate, replicate, and re-emerge as HZ. Finally, Hope-Simpson observed that second episodes of HZ were uncommon, and he hypothesized that the large amount of VZV produced during HZ boosted immunity to VZV, essentially immunizing against another episode of HZ [15]. This observation provided the paradigm for successful vaccination of older adults against HZ. Hope-Simpson’s seminal observations and hypotheses [15] have been confirmed by numerous investigators [18–25].

### Host Immune Responses to Varicella-Zoster Virus

Primary VZV infection (varicella) induces VZV-specific antibodies and cell-mediated immune responses. Varicella-zoster virus-specific antibodies are involved in protecting the host during re-exposure to the virus by contact with persons who have varicella or HZ, but they appear to have little impact on varicella severity, since antibody immunodeficiency (eg, agammaglobulinemia) is not associated with severe varicella or second episodes of varicella [26, 27]. Levels of VZV-specific antibodies remain high with relatively little change during life [28]. Varicella-zoster virus CMI involves T lymphocytes, including CD4^+^ and CD8^+^ effector and memory T cells, as well as natural killer cells [29–31]. The magnitude of VZV-specific T-cell responses are inversely correlated with severity of varicella [6, 32] and HZ [33]. Varicella-zoster virus CMI is necessary to maintain VZV latency.

When latent VZV reactivates, an anamnestic host immune response is usually able to stop VZV replication in time to prevent the development of HZ, and the reactivation is subclinical. Nevertheless, the VZV replication that does occur is sufficient to boost the host’s VZV-specific immune responses [15, 19–21, 23, 25, 34–36]. These contained reversions (Figure 2) maintain immunity to varicella and delay the age-dependent decline in VZV CMI, thus reducing the age-specific incidence of HZ. Similar increases in immunity to VZV are observed during re-exposure to VZV as a result of contact with persons with varicella or HZ [15, 25, 34, 37–47]. When HZ develops, its severity is limited by pre-existing (memory) VZV CMI, but not by antibodies to VZV [33].

### The Role of Latent Varicella-Zoster Virus in Maintaining Host Immunity to Herpes Zoster

On the assumption that exogenous boosting is necessary to maintain VZV-specific immunity and prevent HZ, epidemiological modeling [48, 49] predicted that routine varicella immunization, by markedly reducing the incidence of varicella, would eliminate exposure and thus exogenous boosting and cause an additional 21 million cases of HZ and 5000 additional deaths within 50 years. This prediction precluded the use of varicella vaccine for routine immunization of healthy children in several countries, including England [25, 49].

However, implementation of universal childhood varicella vaccination, which reduced the incidence of varicella (and thus VZV exposure) by >95%, has not resulted in a large increase in the incidence of HZ in the United States. A gradual increase in the age-specific incidence of HZ began well before licensure of varicella vaccine, with no acceleration after implementation of universal childhood varicella vaccination despite a >95% reduction in the incidence of varicella [49–57]. Furthermore, the rate of increase in the age-specific incidence of HZ is comparable in countries with and without childhood varicella vaccination [58–60]. This suggests that exogenous exposure to VZV is not necessary to maintain immunity to VZV and limit the age-specific incidence of HZ. The observation that the incidence of HZ in cloistered monks and nuns who live in the complete absence of contact with children is the same as that in matched controls in the general population provides further evidence that endogenous boosting by contained reversions is sufficient to maintain VZV-specific immunity and prevent HZ [61].

### Herpes Zoster Epidemiology

The lifetime risk of HZ exceeds 30%. The global incidence rate of HZ ranges from 3 to 5/1000 person-years [58] and from 5.23 to 10.9/1000 person-years in persons ≥50 years of age (yoa) [62]; however, these figures are based on data from a few countries only. In the United States, more than 1 million persons develop HZ annually [64, 91]. Both incidence and severity of HZ increase with age, with complications occurring in almost half of older people with HZ [58]; 50% of persons living to 85 yoa will develop HZ [15, 58, 65, 66]. We do not know exactly what distinguishes those who develop HZ from those who do not, but age, immunosenescence, and genetic determinants play significant roles [15, 67–77].

Numerous epidemiologic studies have shown that the age-specific incidence of HZ has increased over the past 7 decades, even in countries where varicella immunization has virtually eliminated varicella [49–57]. This demonstrates that endogenous boosting by contained reversions, rather than exogenous boosting by exposure to VZV (Figure 2), is primarily responsible for maintaining levels of VZV CMI in older adults. The declining incidence of HZ in children (0–18 yoa) and in older adults (≥60 yoa) observed in recent years in the United States is due to prevention of HZ by varicella vaccine (because latent live attenuated Oka VZV (vOka) reactivates less frequently than latent wild-type VZV) and by the introduction of zoster vaccines [55–57, 78].

### Burden of Herpes Zoster and Postherpetic Neuralgia

Herpes zoster and its complications can greatly reduce quality of life (QoL) [79] and result in the irreversible loss of independence. The most common debilitating complication of HZ is postherpetic neuralgia (PHN), characterized by persistence of neuropathic pain and dysesthesia for weeks, months, or even years after the rash has healed [80–84]; the incidence of PHN increases markedly with increasing age [80, 81, 85]. Worldwide, the risk of developing PHN varies from 5% to >30%, depending on study design, age distribution, and definition of PHN [58], with >30% of patients with PHN experiencing persistence of pain for >1 year [58]. Other serious complications of HZ include ophthalmic involvement, neurological complications, and stroke [34, 86]. Herpes zoster is associated with loss of >60 000 QoL years and 2.4 billion dollars in direct medical costs and productivity losses annually in the United States [87].

The persistent neuropathic pain of PHN is often refractory to treatment [88] and can severely compromise QoL and functional status [79]. Individuals with PHN often report disordered sleep, chronic fatigue, weight loss, anorexia, anxiety, and depression. In addition, PHN interferes with the capacity to carry out activities of daily living [79, 84, 89]. The increasing incidence of HZ and its debilitating complications with increasing age provides the impetus to develop vaccines to protect older adults from HZ.

## DEVELOPMENT AND EFFICACY OF CURRENTLY LICENSED ZOSTER VACCINES

Hope-Simpson’s [15] seminal observations and hypotheses, and the development and evaluation by Takahashi and his colleagues of the live attenuated Oka vaccine strain of VZV (vOka) varicella vaccine [18, 25, 92], have led to the development and widespread deployment of safe and effective vaccines against both varicella and HZ.

There are currently 2 licensed zoster vaccines, a live vaccine (zoster vaccine live [ZVL]) based on the attenuated vOka and a recombinant vaccine (recombinant zoster vaccine [RZV]) based on the VZV glycoprotein E (gE) (Table 1). In 2006, the Advisory Committee on Immunization Practices (ACIP) recommended routine administration of ZVL (ZOSTAVAX; Merck) to adults ≥60 yoa for the prevention of HZ and its complications, particularly PHN. However, the superior efficacy of RZV led to its preferred recommendation for immunocompetent adults ≥50 yoa by the ACIP in 2018 [90].

**Table 1.** Herpes Zoster Vaccines Licensed in the United States

Characteristic	ZOSTAVAX (Zoster Vaccine Live; Merck)	SHINGRIX (Recombinant Zoster Vaccine; GlaxoSmithKline)
Vaccine type	Live-attenuated VZV (Oka/Merck); ≥19 400 PFU	Recombinant VZV gE, adjuvanted
Vaccine composition	Two components: 1. lyophilized vaccine 2. sterile diluent	Two components: 1. lyophilized gE antigen 2. AS01B adjuvant suspension
Storage	−50°C to −15°C	+2°C to +8°C
Shelf life	18 months from the date of manufacture of the final filled container when stored at ≤ −15°C	36 months from the date of manufacture when stored at +2°C to +8°C
Dosage and administration	1 dose SQ in deltoid region of upper arm; 0.65 mL/dose	2 doses IM in deltoid region of the upper arm, 2 to 6 months apart; 0.5 mL/dose
Reactogenicity	Low	High
Overall efficacy against incidence of HZ	51.3%	97.2%
Overall efficacy against incidence of PHN	66.5%	91.2%
Persistence of protection against HZ	Up to 8 years	≥10 years (studied up to 10 years)
FDA approval	May 25, 2006 for adults aged ≥60 yoa; March 24, 2011 for adults aged 50–59 yoa	October 20, 2017 for adults aged ≥50 yoaJuly 23, 2021 for adults ≥18 yoa who are or will be at increased risk of HZ due to immunodeficiency or immunosuppression caused by known disease or therapy
ACIP recommendations	For use in immunocompetent adults aged ≥60 years	(1) For use in immunocompetent adults aged ≥50 yoa; (2) For use in immunocompetent adults aged ≥50 yoa who previously received ZOSTAVAX; (3) Preferred over ZOSTAVAX. Should wait at least 8 weeks if previously administered ZOSTAVAX.

Abbreviations: ACIP, Advisory Committee on Immunization Practices; FDA, US Food and Drug Administration; gE, glycoprotein E; HZ, herpes zoster; IM, intramuscular; PFU, plaque-forming units; PHN, postherpetic neuralgia; SQ, subcutaneous; VZV, varicella-zoster virus; yoa, years of age.

### The Challenge of Vaccinating Against Herpes Zoster

Most vaccines, including varicella vaccine, are administered to susceptible persons before infection by the pathogen. These vaccines induce immunity that prevents primary infection and/or disease. In contrast, vaccination against HZ is directed at persons who have previously been infected with VZV and have solid immunity against varicella, but harbor latent VZV that can reactivate and cause HZ [18, 91]. To be effective, zoster vaccine must function as a “therapeutic vaccine” and induce a more potent immune response to prevent reactivation of latent VZV in a person already infected who has pre-existing immunity to VZV.

### Live-Attenuated Oka/Merck Zoster Vaccine (ZVL)

The Oka strain of VZV was isolated from a healthy Japanese child with varicella and attenuated by serial passage at 34°C in human and guinea pig cells by Takahashi and his colleagues [18, 92–94] to produce the live-attenuated Oka vaccine strain of VZV (vOka), which is temperature sensitive. Clinical studies in Japan and subsequently in the United States demonstrated the safety, immunogenicity, and clinical efficacy of vOka in immunocompetent and immunocompromised children and adults [37, 95–107]. This led to licensure of live-attenuated vOka varicella vaccine (VVL) in the United States in 1995 and, eventually, to Centers for Disease Control and Prevention (CDC) recommendation of a routine 2-dose schedule of childhood VVL vaccination in 2007 [25, 108]. Consequently, >90% of healthy US children are now vaccinated, and the incidence of varicella and the number of varicella deaths have decreased by >90% [25, 49, 109–111].

These results set the stage for initiation of a large clinical trial, Department of Veterans Affairs (VA) Cooperative Study number 403: The Shingles Prevention Study (SPS), to test Hope-Simpson’s [15] hypothesis that boosting declining VZV CMI in older adults by vaccination would protect them from HZ and its complications, particularly PHN [91]. Because almost all adults have immunity to varicella, the amount of vOka in zoster vaccine was increased to more than 14 times the amount in varicella vaccine [91].

The SPS was a double-blind, placebo-controlled trial in which 38 546 adults ≥60 yoa were randomized to receive a single subcutaneous dose of high potency live-attenuated Oka/Merck VZV vaccine (ZVL) or placebo at 22 study sites across the continental United States [91].

Zoster vaccine live reduced the “burden of illness due to HZ” (the primary study endpoint, a clinically relevant measure of the severity of illness determined by evaluating the severity of zoster pain over time [112]) by 61.1%; reduced the “incidence of clinically significant PHN” (pain and discomfort due to HZ, scored as ≥3 on a 0–10 scale, persisting for >90 days after rash onset) by 66.5%; and reduced the “incidence of HZ” by 51.3% [91]. Age-specific ZVL efficacy for incidence of HZ was 64% in those 60–69 yoa, but only 37.6% in those ≥70 yoa. Although most of the reduction in the incidence of PHN was due to the reduction in the incidence of HZ in vaccine recipients, ZVL also reduced the proportion of subjects with HZ who developed PHN by >31%, with most of this benefit in the ≥70 yoa group that had the highest risk of developing PHN. Furthermore, ZVL markedly decreased the adverse impact of HZ on QoL and capacity to perform activities of daily living [113].

Although injection site reactions were more frequent among vaccine recipients, they were generally mild. The proportion of study participants reporting serious adverse events (SAEs), and rates of hospitalization and death were comparable in vaccine and placebo recipients [91, 114].

Based upon the results of the SPS, ZVL (ZOSTAVAX) (Table 1) was licensed by the US Food and Drug Administration (FDA) in May 2006 and recommended by the CDC ACIP in October 2006 for routine immunization of adults ≥60 yoa without contraindications for prevention of HZ and its complications, principally PHN [64]. The ACIP recommendation also included administration of ZVL to persons ≥60 yoa regardless of a history of HZ [64]. Two subsequent studies supported this recommendation [115, 116].

A randomized placebo-controlled trial in 22 439 persons 50–59 yoa (the ZOSTAVAX Efficacy and Safety Trial [ZEST] [117]) demonstrated that ZVL efficacy for incidence of HZ was 69.8% during a mean follow-up of 1.3 years. The safety profile was similar to that in the SPS, except that the proportion of injection site reactions was higher (49.5%), reflecting the more robust immune response of younger persons to ZVL [91, 114, 118, 119].

Based upon the ZEST results, the FDA expanded the age indication for ZVL to adults ≥50 yoa. However, the ACIP did not modify their recommendation to administer ZVL to adults ≥60 yoa [120], because of the concern that immunity to HZ would decline in persons vaccinated at 50–59 yoa, leaving them vulnerable to HZ when they were older and the risk and severity of HZ were increased, as well as unfavorable cost-benefit analyses [120]. Zoster vaccine live (ZOSTAVAX) has been removed from the 2021 adult immunization schedule because it is no longer available in the United States [121]. However, ZOSTAVAX continues to be available in most countries, and is likely to continue to be widely available in the future.

#### Persistence of Zoster Vaccine Live Efficacy

Zoster vaccine live efficacy declines over time. The SPS demonstrated persistence of zoster vaccine efficacy for the study endpoints through 4 years postvaccination [91]. Two Persistence Substudies in SPS participants assessed the duration of vaccine efficacy through 11 years postvaccination [122, 123]. Zoster vaccine live efficacy for HZ burden of illness decreased from 61.1% to 37.3%, efficacy for incidence of PHN decreased from 66.5% to 35.4%, and efficacy for incidence of HZ decreased from 51.3% to 21.1%. Vaccine efficacy persisted into year 10 for HZ burden of illness and incidence of PHN and through year 8 for incidence of HZ.

Effectiveness of ZVL against HZ, ophthalmic zoster, and PHN in the general population was demonstrated in several retrospective and observational studies [124–132], with overall vaccine effectiveness (VE) comparable to that observed in the SPS. However, in one large prospective cohort study, VE for incidence of HZ was found to be similar in persons ≥80 yoa and in persons 60–79 yoa [130].

As in the SPS, VE for incidence of PHN was higher than VE for incidence of HZ, suggesting benefit of the vaccine in prevention of PHN beyond prevention of HZ [129–131].

Zoster vaccine live also reduces the severity and duration of PHN and the occurrence of complications, such as ophthalmic zoster, in ZVL recipients who develop HZ. The reduction is greatest for the most severe HZ and for HZ hospitalization [129, 132].

### Recombinant Subunit Herpes Zoster Vaccine (RZV)

A subunit vaccine (RZV, HZ/su) containing recombinant VZV gE and the AS01B adjuvant system was developed by GlaxoSmithKline Vaccines (Wavre, Belgium). Varicella-zoster virus gE is the most abundant glycoprotein in VZV virions and infected cells; it is essential for virus replication and cell-to-cell spread, and it is a major target for VZV-specific CD4^+^ T-cell responses [133, 134]. The liposome-based AS01B adjuvant system contains 2 immunostimulants: (1) monophosphoryl lipid A, a TLR4 agonist that stimulates NF-κB transcription and cytokine production and activates antigen-presenting cells; and (2) QS-21, a natural saponin that promotes antigen-specific antibody and CD4^+^ T-cell responses [135, 136].

Two randomized, placebo-controlled phase III efficacy trials were conducted concurrently in 18 countries in North America, Europe, Asia, Australia, and Latin America to determine the safety and efficacy of RZV against HZ and PHN in adults ≥50 yoa (ZOE-50 [137] and ZOE-70 [138]). Recombinant zoster vaccine efficacy for incidence of HZ was 97.2% in adults ≥50 yoa [137] and 89.8% in adults ≥70 yoa [138]. In an analysis of pooled data from subjects ≥70 yoa in ZOE-50 and ZOE-70 (N = 16 596), RZV efficacy was 91.3% for incidence of HZ and 88.8% for incidence of PHN. In both ZOE-50 and ZOE-70, RZV efficacy for incidence of HZ did not decrease significantly with increasing age and remained high after a mean follow-up of 3.7 years.

In both efficacy trials, RZV was more reactogenic than placebo, with injection site reactions (pain, redness, swelling) and generalized myalgia and fatigue being the most frequent adverse events (AEs) [137–139]. Results of a pooled analysis of safety data from 14 645 RZV and 14 660 placebo recipients in ZOE-50 and ZOE-70 were comparable to results in the individual ZOE trials, with pain being the most frequent solicited local symptom (68.1% in RZV vs 6.9% in placebo recipients, with 3.8% vs 0.2% grade 3). In the 30 days postvaccination, more RZV than placebo recipients reported unsolicited AEs (50.5% vs 32.0%), principally local injection site and systemic reactions. Fatal AEs, SAEs, and potential immune-mediated diseases (IMDs) were comparable in the 2 groups [140].

In October 2017, the FDA approved RZV (SHINGRIX) (Table 1), 2 doses administered intramuscularly 2–6 months apart, for prevention of HZ in adults ≥50 yoa. Subsequently, the ACIP recommended the 2-dose series of RZV (1) for immunocompetent adults ≥50 yoa and (2) for immunocompetent adults previously vaccinated with ZVL; RZV was preferred over ZVL [90]. On July 23, 2021 the FDA approved SHINGRIX for the prevention of HZ in adults ≥18 yoa who are or will be at increased risk of HZ due to immunodeficiency or immunosuppression caused by known disease or therapy [https://www.fda.gov/vaccines-blood-biologics/vaccines/shingrix; accessed 09 August 2021].

An open-label, multicenter study demonstrated that 2 doses of RZV, administered 2 months apart to adults ≥50 yoa with a physician-documented history of HZ, was safe and immunogenic [141], supporting the ACIP recommendation that adults with a history of HZ should receive RZV [90].

In a cohort study among vaccinated and unvaccinated Medicare beneficiaries ≥65 yoa, VE for incidence of HZ was 56.9% and 70.1% for 1 and 2 doses of RZV, respectively. The 2-dose VE was similar in RZV recipients ≥80 yoa or when dose 2 was received ≥180 days after dose 1 [142]. Vaccine effectiveness for PHN was 76.0% and 66.8% for ophthalmic HZ after 2 doses of RZV [142]. The 2-dose RZV VE in patients with immunocompromising chronic conditions, or using immunocompromising drugs, was 64.1% versus 70.9% in immunocompetent patients, and this was not reduced in immunocompetent patients with autoimmune conditions [142].

In a retrospective cohort study in 4 769 819 patients >50 yoa in 2018–2019, 3.6% of whom received 2 doses of RZV and were followed for a median of 7 months, VE for incidence of HZ was 85.5% in RZV recipients 50–79 yoa. Vaccine effectiveness for incidence of HZ in RZV recipients ≥80 yoa was 80.2%, compared with 89.8% in ZOE 70 [143].

#### Persistence of Recombinant Zoster Vaccine Efficacy

A number of phase I, I/II, and II studies [134, 144–146] established that 2 doses of RZV, administered 1 or 2 months apart, were well tolerated and induced much greater VZV-specific and VZV gE-specific CD4^+^ T-cell and antibody responses than ZVL in those ≥50 yoa [134]. These results are mirrored by side-by-side comparison of ZVL and RZV immunological responses, showing that immunization with RZV stimulates stronger VZV- and gE-specific CMI and humoral responses than ZVL [147, 148]. Anti-gE antibody concentrations and VZV gE-specific CMI responses, measured by enumerating CD4^+^ T cells expressing at least 2 of 4 activation markers (interferon-γ, interleukin-2, tumor necrosis factor [TNF]-α, CD40 ligand) [134], persisted through year 6 postvaccination [146, 149]. At year 9 postvaccination, median gE-specific CD4^+^ T-cell responses and median anti-gE antibody concentration both remained at the same level as year 6 postvaccination, which was 3.4 times and 7.4 times higher, respectively, than prevaccination levels [150]. Ten years postvaccination, median levels of CD4^+^ T cells and VZV gE antibody were 3.3-fold and 5.9-fold above prevaccination levels [151, 152]. However, persistence of CMI responses did not appear to be sustained in at least one quarter of the subjects in the older age group (see Figure 1C in [150]), and both cell-mediated and humoral immune responses declined appreciably in all RZV recipients during the first 4 years postvaccination [150, 152], indicating that a booster dose of RZV may be required, especially in older individuals, to assure continued protection.

## BOOSTER VACCINATIONS

In an attempt to reverse waning ZVL efficacy by boosting VZV CMI, ZVL was administered to 201 adults ≥70 yoa who had received ZVL a decade previously and to 199 age-matched HZ history-negative adults who had never received ZVL. Varicella-zoster virus-specific CMI responses of recipients of a second (booster) dose of ZVL were greater before vaccination than the VZV-specific CMI responses of the first-time vaccinees, and that difference persisted 1 year postvaccination [153, 154].

Two doses of RZV, given to persons ≥65 yoa who had received ZVL ≥5 years previously, were safe and elicited increases in anti-gE antibody concentrations and gE-specific CD4 T-cell frequencies, as well as activation marker profiles that were comparable to those in ZVL-naive RZV recipients, and persisted for at least 12 months after RZV dose 2 [155, 156]. Although intervals of <5 years between ZVL and RZV were not examined, there is no reason why RZV should be less safe or immunogenic when administered <5 years after ZVL. Accordingly, the ACIP recommends administration of RZV to adults ≥50 yoa whether or not they have received ZVL, but no sooner than 8 weeks after ZVL [90].

A cohort study among vaccinated and unvaccinated Medicare beneficiaries ≥65 yoa who had received ZVL during the previous 5 years showed that the 2-dose RZV VE for incidence of HZ was only 63.0%, reflecting the protective effect of ZVL in the RZV unvaccinated comparator group [142].

Safety and immunogenicity of 2 doses of RZV, administered 10 years after the initial 2-dose RZV vaccination, were evaluated in a cohort of 62 individuals. Both gE-specific antibody titers and gE-specific CD4^+^ T-cell counts increased substantially, to even higher levels than elicited by the initial RZV vaccination [152]. However, a single booster dose of RZV was sufficient; the second RZV booster dose did not increase levels of immunity to VZV over those elicited by the first [152].

## ZOSTER VACCINES IN IMMUNOCOMPROMISED POPULATIONS

Although antiviral treatments are available for HZ, prevention of HZ by vaccination is of importance to immunocompromised patients because they are at increased risk for HZ and severe complications [157, 158], and even early antiviral therapy does not prevent the development of PHN. The ACIP recommends administration of ZVL or RZV to persons taking low-dose immunosuppressive therapy (eg, <20 mg/day prednisone or equivalent or using inhaled or topical steroids), persons anticipating immunosuppression, and patients who have recovered from an immunocompromising illness [90]. Immunocompromised persons and those on moderate to high doses of immunosuppressive therapy were excluded from the SPS and ZOE-50/70 efficacy trials [91, 137, 138]. On July 23, 2021 the FDA approved SHINGRIX for the prevention of HZ in adults ≥18 yoa who are or will be at increased risk of HZ due to immunodeficiency or immunosuppression caused by known disease or therapy [https://www.fda.gov/vaccines-blood-biologics/vaccines/shingrix; accessed 09 August 2021].

### Zoster Vaccine Live

Zoster vaccine live (ZVL), like varicella vaccine and other live-attenuated vaccines, is contraindicated in immunocompromised patients because of the theoretical risk that attenuated vOka might cause serious disease. However, >95% of adults in the United States and most other developed countries will have experienced varicella in childhood and thus are likely to have residual VZV CMI, which would limit the risk of AEs caused by replication and dissemination of vOka [158, 159]. Despite recommendations to the contrary, ZVL has been administered to many immunocompromised adults in the course of routine vaccination [125, 159–168], with few SAEs.

Zoster vaccine live was safe and immunogenic in patients ≥18 yoa with end-stage renal disease who received ZVL before renal transplant [169]. Zoster vaccine live reduced incidence of HZ by 51% in patients ≥60 yoa with end-stage renal disease on dialysis [170], similar to VE in immunocompetent individuals of the same age, but was significantly greater among patients on dialysis for <2 years than among patients on dialysis longer (72% vs 36%, respectively) [170].

Because ZVL is contraindicated in immunocompromised persons, zoster vaccines containing vOka inactivated either by heat (ZVHT) or γ-irradiation (ZVIN) were developed and administered to immunocompromised adults in clinical trials [171–178] (Supplementary Table 1) to determine their safety, immunogenicity, and efficacy. Both vaccine formulations were generally well tolerated, but immune responses were variable, depending on the underlying conditions and treatments. The ZVHT did not elicit a humoral response in recipients of autologous or allogeneic hematopoietic stem-cell transplants (HSCT), and T-cell responses were poor in allogeneic HSCT recipients [173]. The ZVIN efficacy in autologous HSCT recipients and patients with solid tumors was comparable to that of ZVL for incidence of HZ [176, 177] and PHN (83.7%) [176], but there was no reduction in incidence of HZ in patients with hematologic malignancies [177, 178].

### Recombinant Zoster Vaccine

Recombinant zoster vaccine (RZV) has been evaluated in clinical trials involving persons with a variety of immunocompromising conditions [179–185] (Supplementary Table 1) and has been administered to immunocompromised persons in the course of routine vaccination [142].

Recombinant zoster vaccine was safe and immunogenic in patients undergoing autologous HSCT [179], in patients with solid tumors before or during chemotherapy [183], in patients with hematologic malignancies during or after immunosuppressive therapy [182], and in chronically immunosuppressed renal transplant recipients [184]. Humoral and cell-mediated immune responses persisted for at least 1 year after vaccination [179, 182–184]. Two doses of RZV, administered between 9 and 24 months after transplant, were safe and did not increase the incidence of graft-versus-host disease (a potential concern due to the potent AS01B adjuvant) in allogeneic HSCT recipients [186].

Vaccine efficacy for incidence of HZ was 87.2% during a median follow-up of 11.1 months in patients with hematologic malignancies [182]. A randomized phase III trial in 1846 patients who had undergone a recent autologous HSCT (ZOE-HSCT) showed RZV efficacy for incidence of HZ of 68.2% during a median follow-up of 21 months [181], comparable to ZVL efficacy (63.8%) in a similar population [176]. Recombinant zoster vaccine also reduced the adverse impact of HZ on the QoL of these HSCT recipients [185].

## VACCINATION OF PERSONS INFECTED WITH HUMAN IMMUNODEFICIENCY VIRUS

Persons infected with human immunodeficiency virus (HIV) are at much higher risk of HZ, HZ recurrence, and HZ complications than the general population [157, 187]. Herpes zoster complications are approximately 3 times more frequent in persons with HIV infection than in age-matched members of the general population [180]. Neither ACIP nor the World Health Organization (WHO) guidelines currently include a recommendation for HZ vaccination in persons infected with HIV, even with CD4 T-cell counts ≥200 cells/μL [188]. The Infectious Diseases Society of America (IDSA) guidelines for immunocompromised individuals include a recommendation to administer ZVL to asymptomatic HIV-infected persons ≥60 yoa who are VZV-seropositive with CD4 T-cell counts ≥200 cells/μL [189]. Likewise, the British HIV Association guidelines recommend administration of ZVL to asymptomatic HIV-infected persons ≥70 yoa who are VZV-seropositive with CD4 T-cell counts ≥200/cells/μL [190]. A recent update to the HIV Primary Care Guidelines from the HIV Medicine Association of the IDSA recommends administration of 2 doses of RZV to HIV-infected persons ≥50 yoa with CD4 T-cell counts ≥200 cells/μL [191].

### Zoster Vaccine Live

In the United States, ZVL is contraindicated in HIV-infected persons with CD4^+^ T-cell counts <200 cells/μL [189], because of the theoretical risk that attenuated vOka might cause serious disease. In HIV-infected adults with CD4^+^ T-cell counts of >400 cells/μL and HIV viral loads <1000 copies of HIV RNA/mL, 2 doses of VVL (Varivax; Merck) were well tolerated, but only moderately immunogenic [192]. Four doses of a heat-inactivated Oka/Merck zoster vaccine (ZVHT) induced low, but significant, VZV-specific T-cell and antibody responses and had a favorable safety profile in HIV-infected adults with ≥200 CD4^+^ T cells/μL [173].

Two doses of ZVL given 6 weeks apart to VZV-seropositive HIV-infected adults ≥18 yoa on antiretroviral therapy (ART) with CD4^+^ T-cell counts ≥200/μL and viral loads <75 copies of HIV RNA/mL were safe and immunogenic [193].

Despite evidence of safety and immunogenicity, ZVL uptake among persons infected with HIV has been low. This is due, at least in part, to the lack of clear guidelines [194, 195]. A study of ZVL uptake in HIV-infected Veterans ≥50 yoa on ART with CD4 T-cell counts ≥200/μL showed that vaccination rates increased steadily between 2006 and 2015, but the cumulative vaccination rate was less than half that in uninfected Veterans [196].

### Recombinant Zoster Vaccine

A double-blind placebo-controlled phase I/IIa trial of RZV was carried out in 3 cohorts of HIV-infected persons ≥18 yoa: 94 ART recipients with high (≥200/μL) CD4^+^ T-cell counts, 14 ART recipients with low (50–199/μL) CD4^+^ T-cell counts, and 15 ART-naive adults with high (≥500/μL) CD4^+^ T-cell counts [180]. A 3-dose regimen of RZV was safe and immunogenic in all 3 cohorts, with humoral and cell-mediated immune responses persisting for at least 1 year after the third RZV dose. In addition, 1 year after RZV dose 3, VZV gE-specific CD4^+^ T-cell responses were higher than those after natural HZ. There were no vaccine-related SAEs and no impact of RZV on HIV viral load or CD4^+^ T-cell counts [180].

## VACCINATION OF PERSONS WITH IMMUNE-MEDIATED DISEASES

For patients with IMDs, the general recommendation is to vaccinate before initiating immunosuppressive therapy. The rationale is 2-fold. (1) Compared with the general population, patients with autoimmune diseases such as rheumatoid arthritis (RA), inflammatory bowel disease (IBD), and systemic lupus erythematosus (SLE) are at increased risk for HZ [197] and would benefit from prophylactic immunization. (2) There is concern that the immunosuppressive medications used in these patients place them at even greater risk of HZ [159]. For example, tofacitinib, an immunomodulatory drug that inhibits Janus kinases, doubles the risk of HZ in RA patients receiving anti-TNF and other biologics [198].

### Zoster Vaccine Live

Several retrospective studies have shown that ZVL is safe and reduces the risk of HZ in patients with autoimmune diseases, including those exposed to biologic agents [161, 163, 199, 200].

Zoster vaccine live was generally safe and immunogenic in a trial with RA patients on methotrexate who received tofacitinib 2–3 weeks after ZVL [201], with SAEs in 3 of 55 tofacitinib recipients (5.5%). One SAE consisted of varicella in a patient who lacked pre-existing immunity to VZV. Follow-up for 27 months postvaccination could not assess long-term protection against HZ, due to the small number of participants [202].

A randomized, placebo-controlled trial evaluating the safety, immune response, and effectiveness of ZVL in 617 adults ≥50 yoa with autoimmune diseases receiving anti-TNF-α therapy observed no varicella-like or HZ-like rashes caused by vOka during the 6-week risk period following ZVL administration, indicating that ZVL is safe in this population [203]

Zoster vaccine live was safe in a small pilot study in patients with SLE, with no SAEs or disease flares during 12 weeks of follow-up [204]. A randomized, placebo-controlled phase IV trial in patients with SLE demonstrated ZVL’s safety and immunogenicity [205].

### Recombinant Zoster Vaccine

When administering RZV to patients with IMDs, there is a theoretical risk that adjuvants such as AS01B might induce or exacerbate autoimmune diseases [206].

In the ZOE-50/70 population, exacerbations of existing IMDs were comparable in RZV and placebo recipients: 16 RZV (1.6%) and 23 (2.4%) placebo recipients reported new onset of a different IMD [140]. Recombinant zoster vaccine efficacy for incidence of HZ in the pooled ZOE-50/70 population with a pre-existing IMD was 90.5%, and incidence of SAEs was similar in RZV and placebo recipients [207]. However, the ZOE-50/70 trials excluded individuals who had received immunosuppressive or immune-modifying drugs for >15 consecutive days within 6 months before the first dose of RZV, or those who had received prednisone ≥20 mg/day or equivalent.

In a cohort study in RZV vaccinated and unvaccinated Medicare beneficiaries ≥65 yoa, VE for incidence of HZ in persons with autoimmune diseases was similar to that in the overall population [142].

Retrospective studies of patients in 2 rheumatology outpatient centers identified 762 patients with systemic rheumatic diseases who received RZV [208, 209]. Disease flares, with onset up to 12 weeks after an RZV dose, occurred in 6.7% [208] and 16.4% [209], with the majority occurring after the first RZV dose—perhaps because patients experiencing a flare after RZV dose 1 declined dose 2 [208]. Nine patients experienced flares after both doses of RZV. Rheumatoid arthritis patients had the highest flare rate in both studies. In the first study [208] the authors stated that the rate of flares in patients receiving RZV was lower than the background incidence of RA disease flares observed in their registry. In the second study [209], only the use of glucocorticoids at the time of RZV administration was significantly associated with flares. Flares in 18 of 59 patients coincided with a change in rheumatic disease treatment at the time of RZV administration. The authors concluded that it may be preferable to give RZV to patients with IMDs when their disease is quiescent. RZV was generally well tolerated in both studies, with mild local and systemic AEs in 12.7% and 8.5% of patients, respectively.

A prospective study [210] followed 67 patients with irritable bowel disease (IBD) for a median of 207 days after each of 2 doses of RZV. The AEs were similar to those observed in the ZOE-50/70 efficacy trials [137, 138], and there were no cases of HZ. One patient with ulcerative colitis suffered a major flare 3 days after RZV dose 2 [210].

In the ZOE-50/70 trials, 27 RZV and 8 placebo recipients reported an episode of gout or gouty arthritis during the 30-day post-vaccination period [211]. Of the 35 affected persons, 19 RZV and 3 placebo recipients reported their first episode of gout, whereas 8 RZV and 5 placebo recipients experienced a flare of pre-existing gout [212]. A retrospective study of patients in a rheumatology clinic reported that 18% of patients with gout experienced a flare within 12 weeks after receiving RZV [209]. Vaccines other than RZV have also been associated with an increased risk of flares of gout [213].

## POSTLICENSURE SAFETY

### Zoster Vaccine Live

A review of worldwide safety data during the first 10 years (2006 to 2016) after ZVL (ZOSTAVAX) licensure, with >34 million doses distributed [214], concluded that the safety profile was comparable to the safety data from efficacy trials and postlicensure studies. A recent review of ZVL safety in adults 70–79 yoa using an electronic database of participating primary care practices in Australia [215] did not identify any new safety concerns.

In 2014, Tseng et al [216] reported the first laboratory-documented case of HZ caused by ZVL in a 68-year-old healthy female diagnosed with HZ 10 months after receiving ZOSTAVAX. Although the patient had spent her entire life in the United States and thus likely had experienced varicella, past primary infection with VZV was not confirmed. This report may represent a rare case of HZ caused by vOka in a person who was VZV-naive when she received ZVL. A 49-year-old renal transplant recipient developed an extensive skin rash caused by vOka 3 weeks after receiving ZVL [217]. The patient was treated successfully with intravenous acyclovir.

There have been few reports of death from disseminated vOka following ZVL. Two cases of fatal disseminated vOka after administration of ZVL have been described in persons with chronic lymphocytic leukemia [218, 219]. A third fatal case of disseminated vOka following ZVL occurred in a patient with cardiovascular disease, chronic obstructive pulmonary disease (COPD), and RA [220]. His medications included low-dose corticosteroids and low-dose methotrexate.

### Recombinant Zoster Vaccine

Postlicensure safety monitoring of RZV [221] in the Vaccine Adverse Events Reporting System (VAERS) during the first 8 months of use, with approximately 3.2 million RZV doses distributed, showed a safety profile consistent with that observed in prelicensure efficacy trials [137, 138].

There have been a few reports of SAEs, including several cases of HZ, after administration of RZV (SHINGRIX), both in immunocompetent and in immunocompromised individuals [222–229]. Although there was a temporal correlation between these events and receipt of RZV, causality was not established.

In a large postmarketing study using the Medicare claims database, the FDA, the Centers for Medicare and Medicaid Services, and the CDC investigated the risk of Guillain-Barré Syndrome (GBS) after administration of SHINGRIX [230]. The FDA determined that there was an association of GBS with SHINGRIX, but that available evidence was insufficient to establish a causal relationship. The FDA concluded that a warning about GBS should be included in the Warnings and Precautions section of the Prescribing Information for SHINGRIX [230], and GlaxoSmithKline has amended it accordingly [231].

## CONCOMITANT ADMINISTRATION OF ZOSTER VACCINES WITH OTHER VACCINES

### Zoster Vaccine Live (ZOSTAVAX)

A major impediment to administration of the portfolio of vaccines recommended for older adults is their low frequency of encounters with healthcare providers. Thus, it is important to administer more than 1 vaccine during a single visit. The FDA and ACIP have routinely endorsed the safety and efficacy of concomitant administration of live-attenuated and inactivated vaccines at separate injection sites [232].

A clinical trial [233] demonstrated that concomitant administration of ZVL (ZOSTAVAX) and Pneumovax23 resulted in lower VZV antibody titers than observed when ZVL was administered 4 weeks after Pneumovax23; cellular immunity to VZV was not evaluated. Despite evidence that VZV-specific CMI and not antibody to VZV is necessary to protect against HZ, the ZOSTAVAX prescribing information (package insert) was revised in 2009 [http://wayback.archive-it.org/7993/20170722150959/https://www.fda.gov/BiologicsBloodVaccines/Vaccines/ApprovedProducts/ucm195993.htm; accessed 07 September 2021], advising against concomitant administration of the 2 vaccines. This likely resulted in numerous missed opportunities for vaccination [234].

Two cohort studies [235, 236] comparing ZVL VE for incidence of HZ in eligible adults receiving concomitant versus sequential vaccination with Pneumovax23 and ZOSTAVAX provided direct evidence that concomitant administration did not reduce ZVL effectiveness. Nevertheless, the ZOSTAVAX prescribing information still advises providers to consider administering the 2 vaccines ≥4 weeks apart [237].

Safety and immunogenicity were comparable in adults ≥50 yoa vaccinated with ZVL (ZOSTAVAX) and trivalent inactivated influenza vaccine (IIV3) concomitantly and ≥4 weeks apart [238]. Likewise, concomitant administration of ZVL and quadrivalent inactivated influenza vaccine (IIV4) to subjects ≥50 yoa was well tolerated, and immunogenicity was comparable to that observed when either vaccine was administered alone [239].

### Recombinant Zoster Vaccine (SHINGRIX)

A trial assessing safety and immunogenicity of concomitant administration of RZV and Pneumovax23 to adults ≥50 yoa [240] revealed no immunologic interference. No safety concerns were identified.

A trial evaluating safety and immunogenicity of coadministration of the first dose of RZV with reduced-antigen-content diphtheria-tetanus-acellular pertussis vaccine (Tdap) in adults ≥50 yoa [241] demonstrated noninferiority for all vaccine antigens, except pertussis pertactin. However, because pertactin-specific immune responses after coadministration of RZV and Tdap compared favorably with protective levels in a German household contact study [242], these data do not suggest clinically relevant interference between RZV and Tdap. No safety concerns were identified.

A phase III trial in adults ≥50 yoa evaluating coadministration of RZV and unadjuvanted quadrivalent influenza vaccine (IIV4) [243] demonstrated noninferiority of antibody responses to both vaccines compared with sequential administration. There was no decrease in immunogenicity, and no safety concerns were noted [243]. There are currently no recommendations for concomitant administration of MF59-adjuvanted influenza vaccine and RZV, because concomitant administration of 2 adjuvanted vaccines has not been evaluated [244].

## GLOBAL USE OF ZOSTER VACCINES

Zoster vaccine live (ZOSTAVAX) and RZV (SHINGRIX) are currently licensed or distributed in 62 countries worldwide (Supplementary Table 2). Although both vaccines are approved in all 28 countries within the European Union (EU), only 9 include recommendations for vaccination against HZ in their national vaccination policies [245–250].

### Zoster Vaccine Live

Zoster vaccine live (ZOSTAVAX) is licensed in 57 countries and is available for the prevention of HZ and its complications in 41 (Merck, written personal communication, March 11, 2021) (Supplementary Table 2). As of February 2021, 51 million doses of ZVL have been distributed globally (30.5 million within the United States; 20.5 million in other countries). In 2020 alone, 2.2 million doses of ZOSTAVAX were distributed, with >90% of the total in Italy, United Kingdom, Greece, Australia/New Zealand, and Korea (Merck, personal communication).

In 2016, Ontario was the first Canadian province to publicly fund ZVL for adults 65–70 yoa [215, 251]. In November 2016, ZVL was provided by Australia’s National Immunization Program without charge to the cohort of adults 70 yoa, with catch-up for those 71–79 yoa. The program is funded until October 2021 [215].

In March 2016, Japan extended approval of VVL (BIKEN), which has a higher titer of vOka than ZVL (ZOSTAVAX), for the prevention of HZ in individuals ≥50 yoa [252–254].

Live-attenuated zoster vaccine NBP608 (SKY Zoster), manufactured with vOka by SK Bioscience (Andong-si, Republic of Korea), was approved by the Korean Ministry of Food and Drug Safety in October 2017. A phase III trial demonstrated that NBP608 was safe and immunogenic and noninferior to ZOSTAVAX in healthy adults ≥50 yoa [255].

### Recombinant Zoster Vaccine

Recombinant zoster vaccine (SHINGRIX) has been approved in 35 countries and is available in 8 countries (including the US) (GlaxoSmithKline, written personal communication, February 8, 2021) (Supplementary Table 2). In 2018, the Canadian National Advisory Committee on Immunization updated its HZ vaccine recommendations to provide a preferential recommendation for RZV over ZVL [256]. However, the recommendation still remains that ZVL may be considered for immunocompetent persons in whom RZV is contraindicated, unavailable, or inaccessible.

Recombinant zoster vaccine (SHINGRIX) was approved in Europe (EU countries) and Japan in March 2018, in China in May 2019, in New Zealand in Jan 2020, in Hong Kong in October 2020, and in Singapore in January 2021. Austria recommends administering 2 doses of SHINGRIX at least 2 months apart to all adults >50 yoa, independent of immune status. In severely immunocompromised persons, off-label administration of RZV may be considered. However, RZV is not currently available in Austria, but it may be ordered through pharmacies in other European countries [248]. The German Standing Committee on Vaccination recommends 2 doses of SHINGRIX 2 to 6 months apart in the following: healthy adults ≥60 yoa; immunocompromised adults ≥50 yoa; and adults ≥50 yoa with underlying diseases such as diabetes, RA, IBD, COPD, and asthma [257]. The difficulty producing large amounts of SHINGRIX, the requirement for 2 doses, and the local and systemic AEs are significant barriers to the universal adoption of RZV.

## OTHER ZOSTER VACCINES

Both currently licensed vaccines have disadvantages: ZOSTAVAX efficacy for HZ incidence is low, compared with SHINGRIX, and the efficacy wanes over several years. In addition, as a live-attenuated vaccine, it cannot be used in severely immunocompromised patients. On the other hand, SHINGRIX requires 2 doses, separated by 2–6 months, and the potent AS01B adjuvant makes SHINGRIX more reactogenic. Furthermore, the association of GBS with SHINGRIX is of concern, although at present it is unclear if there is a causal relationship. In addition, due to significant supply shortages, most countries where SHINGRIX is licensed have yet to administer it. Thus, the development of further zoster vaccines, either based on ZVL and RZV, or on different vaccine platforms, is being pursued. Two clinical trials to assess safety, tolerability, and immunogenicity of different formulations of adjuvanted VZV gE subunit vaccines are currently registered in the United States (ClinicalTrials.gov Identifier NCT03820414 and NCT04210752). However, results are not yet available. A placebo-controlled trial to assess safety and efficacy of a live-attenuated VZV vaccine in healthy adults ≥40 yoa is currently registered in China (ClinicalTrials.gov Identifier NCT04334577), but enrollment has not yet started.

The recent findings of safety and impressive efficacy of 2 messenger ribonucleic acid (mRNA)-lipid vaccines for severe acute respiratory syndrome coronavirus 2 that were granted Emergency Use Authorization by the FDA may lead to new mRNA vaccines for other diseases, including HZ [258, 259]. In a nonhuman primate model, an mRNA-lipid vaccine expressing VZV gE induced gE-specific antibody and CD4^+^ T-cell responses comparable to RZV and superior to those induced by ZVL [260]. A different adjuvanted formulation with a single-stranded RNA derived from the cricket paralysis virus is also in the early stages of development [261]. Several other zoster vaccines are also in development (see Table 2).

**Table 2.** Other Zoster Vaccines and Zoster Vaccine Candidates

Company Name	Country	Vaccine Name	Characteristics	Status	Reference
SK Bioscience	South Korea	SKY Zoster (NBP608)	Live-attenuated Oka/SK strain	NCT03120364 Phase 3 (completed); approved in 2017 in Korea for adults ≥50 yoa	Choi WS, Choi JH, Jung DS, et al [255]
Curevo/GC Pharma/IDRI	USA/South Korea	CRV-101	gE subunit vaccine with proprietary adjuvant	NCT03820414 Phase 1 (completed)	https://curevovaccine.com/2020/09/curevo-vaccine-announces-robust-antibody-response-results-of-phase-i-clinical-trial-of-investigational-vaccine-for-shingles-crv-101/
EyeGene/Novotech	South Korea/Australia	EG-HZ	Adjuvanted recombinant VZV gE protein	NCT04210752 Phase 1 (completed)	http://eyegene.co.kr/ eng/product_pipeline/EG_HZ/?lang = en_US
BCHT Biotechnology	China	Zoster Vaccine, Live	Live-attenuated Oka VZV vaccine	NCT04334577 Phase 3 (not yet recruiting)	https://ichgcp.net/clinical-trials-registry/ NCT04334577
Vaccitech/ CanSino Biologics	United Kingdom/Hong Kong	VTP-400 (CSB016)	Adenoviral vaccine (ChAdOx1) encoding VZV gE	Preclinical	www.vaccitech.co.uk/pipeline/
GeneOne Life Science	South Korea	GLS-5100	plasmid containing VZV-derived gene encoding a VZV protein; administered to the body using electroporation	Preclinical	www.genels.com/en/sub/technology/ vaccine.asp
Akshaya Bio	Canada	Chimigen ShingVax	recombinant proteins, antigens fused to the Fc fragment of a murine monoclonal antibody through proprietary peptide linkers.	Preclinical	www.akshayabio.com/technology.html
Merck/Moderna	USA	VZV gE mRNA/LNP	mRNA expressing truncated VZV gE protein in lipid nanoparticles	Nonhuman primate studies	Monslow MA, Elbashir S, Sullivan NL, et al [260]
CPL Biologicals	India	VZV Vaccine	Based on nanoparticle technology from Novavax (will target varicella and HZ)	In development	http://cplbio.com/rd/rd-pipeline/

Abbreviations: gE, glycoprotein E; HZ, herpes zoster; IDRI, Infectious Disease Research Institute; LNP, lipid nanoparticle(s); mRNA, messenger ribonucleic acid; VZV, varicella-zoster virus; yoa, years of age.

## GLOBAL NEEDS FOR A ZOSTER VACCINE

The world’s population is aging [262]. One in 6 persons worldwide and one in 4 in North America and Europe will be ≥65 yoa by 2050 [263]. The population of older adults is expected to double between 2019 and 2050 in every region except sub-Saharan Africa [263].

One third of adults will develop HZ in their lifetime, and half who reach 85 yoa will be afflicted with HZ [15, 58, 65, 66, 264]. Although rarely fatal, HZ has a major adverse impact on the elderly, causing chronic neuropathic pain, decreased physical and social functioning, emotional distress, reduced productivity, medical costs, and irreversible loss of independence [89, 265, 266].

We estimate that, in the absence of HZ vaccination, 278 million cases of HZ will occur globally over the next decade, including 10.5 million cases in persons ≥85 yoa; 20.7 million persons ≥50 yoa would experience PHN (based on population projections [267] and published incidence rates for HZ and PHN [15, 268]).

Herpes zoster vaccination is cost effective in older adults [268, 269], but low- and middle-income countries (LMICs) have many other public health priorities. Nevertheless, because childhood varicella vaccination would eventually reduce the incidence of HZ in older adults, adding it to the WHO Regional Strategic Plan for Immunization for the African region [270] would eventually reduce the human and economic cost of HZ. Furthermore, as the current population of persons already latently infected with wild-type VZV ages, the burden of HZ among older adults will increase enormously. Thus, a safe, effective, and inexpensive HZ vaccine is needed.

The Oka vaccine strain of VZV is attenuated with respect to the consequences of primary infection, but, similar to wild-type VZV, it establishes latent infection and can reactivate to cause HZ [25, 271–275]. However, the incidence of HZ caused by vOka in vaccinated children is much lower than the incidence of HZ after varicella caused by wild-type VZV. Thus, the Oka vaccine strain of VZV appears to be attenuated with respect to reactivation [25, 274, 275].

Unlike vOka, nonreplicating VZV vaccines such as RZV and others (Table 2) are inherently safe in immunodeficient patients, including persons in LMICs with endemic diseases such as malaria, tuberculosis, and HIV/acquired immune deficiency syndrome [276–281]. Thus, these vaccines would constitute ideal candidates for safe varicella and zoster vaccines. However, because they do not establish latency, the genetic information for the VZV antigens they encode does not persist, and, consequently, they may fail to induce long-term immunity because there is no endogenous boosting of VZV CMI by contained reversions (see background, The Role of Latent VZV in Maintaining Host Immunity to HZ).

Bacillus Calmette-Guérin (BCG) vaccines provide a vaccine platform [282, 283] that could serve the public health needs of LMICs throughout the world. Bacillus Calmette-Guérin has many advantages as a multivalent vaccine platform ideally suited for LMICs. The genome is large enough to accommodate more than 20 different antigens, providing a platform for a single vaccine against many different diseases. Low- and middle-income countries already have experience producing and distributing BCG. It can be easily and inexpensively produced in large quantities, it can be rendered incapable of replication and thus much safer, and it can be self-administered via an intradermal microneedle patch [284], without need for a cold chain, taking advantage of the anamnestic immune response of the millions of persons in LMICs who have already received BCG.

## Supplementary Data

Supplementary materials are available at *The Journal of Infectious Diseases* online. Supplementary materials consist of data provided by the authors that are published to benefit the reader. The posted materials are not copyedited. The contents of all supplementary data are the sole responsibility of the authors. Questions or messages regarding errors should be addressed to the author.

jiab387_suppl_Supplementary-Material

jiab387_suppl_Supplementary-Table-1

jiab387_suppl_Supplementary-Table-2

## Notes

***Acknowledgments.*** This review is dedicated to the memory of Edgar Hope-Simpson, whose seminal observations and prescient hypothesis on herpes zoster provided the inspirational and rationale for live-attenuated Oka herpes zoster vaccine, and Michiaki Takahashi, whose development of live-attenuated Oka varicella-zoster virus has made it possible to virtually eliminate varicella and markedly reduce the adverse impact of herpes zoster on the growing population of older adults who are at increased risk. We thank the VA San Diego Healthcare System Research Service and the Veterans Medical Research Foundation, San Diego for their administrative and other non-monetary support, and Donna Mussatto and Mark Ashbaugh for their invaluable expertise and assistance in preparing this manuscript.

***Financial support.*** R. H. and M. N. O. are supported, in part, by the Veterans Affairs (VA) Cooperative Studies Program, the James R. and Jesse V. Scott Fund for Shingles Research, and private donations. J. I. C. is supported by the Intramural Research Program of the National Institute of Allergy and Infectious Diseases.

***Supplement sponsorship.*** This supplement is sponsored by the Bill and Melinda Gates Foundation.

***Potential conflict of interests.*** J. I. C. is named as an inventor on a patent application for a live attenuated varicella vaccine and has a Collaborative Research and Development Agreement with GlaxoSmithKline involving an Epstein-Barr virus vaccine. M. N. O. has served as consultant to GlaxoSmithKline and as a member of the Data Safety Monitoring Board for a National Institutes of Health-funded clinical trial of ZOSTAVAX. All authors have submitted the ICMJE Form for Disclosure of Potential Conflicts of Interest. Conflicts that the editors consider relevant to the content of the manuscript have been disclosed.
