# Supplementary material for: Herpes Zoster Vaccines
Source: J Infect Dis. 2021 Sep 30;224(Suppl 4):S429–42. doi: 10.1093/infdis/jiab387 (PMC8482024; doi:10.1093/infdis/jiab387)
Supplement: jiab387_suppl_Supplementary-Material [file jiab387_suppl_supplementary-material.docx]

1. Centers for Disease Control and Prevention. Varicella Updated December 2020. In: Hamborsky J, Kroger A, Wolfe C, eds. Epidemiology and prevention of vaccine-preventable diseases (The Pink Book). 13th ed, **2015**.

2. Levin MJ, Schmader KE, Oxman MN. Varicella and herpes zoster. In: Kang S, Amagai M, Bruckner AL, et al., eds. Fitzpatrick's dermatology. 9th ed. New York, NY: McGraw-Hill Education, **2019**.

3. Weller TH. Varicella and herpes zoster. Changing concepts of the natural history, control, and importance of a not-so-benign virus. N Engl J Med **1983**; 309:1362-8.

4. Gershon AA, Breuer J, Cohen JI, et al. Varicella zoster virus infection. Nat Rev Dis Primers **2015**; 1:15016.

5. Liyanage NP, Fernando S, Malavige GN, et al. Seroprevalence of varicella zoster virus infections in Colombo district, Sri Lanka. Indian J Med Sci **2007**; 61:128-34.

6. Malavige GN, Jones L, Kamaladasa SD, et al. Viral load, clinical disease severity and cellular immune responses in primary varicella zoster virus infection in Sri Lanka. PLoS One **2008**; 3:e3789.

7. Hastie IR. Varicella-zoster virus affecting immigrant nurses. Lancet **1980**; 2:154-5.

8. Nassar NT, Touma HC. Brief report: susceptibility of Filipino nurses to the varicella-zoster virus. Infect Control **1986**; 7:71-2.

9. Gallagher J, Quaid B, Cryan B. Susceptibility to varicella zoster virus infection in health care workers. Occup Med (Lond) **1996**; 46:289-92.

10. Arvin AM, Gilden D. Varicella-zoster virus. In: Knipe DM, Howley PM, eds. Fields virology. 6th ed. Vol. 2. Philadelphia: Lippincott Williams & Wilkins, **2013**:2015-57.

11. Gershon AA, Chen J, Davis L, et al. Latency of varicella zoster virus in dorsal root, cranial, and enteric ganglia in vaccinated children. Trans Am Clin Climatol Assoc **2012**; 123:17-35.

12. Chen JJ, Gershon AA, Li Z, Cowles RA, Gershon MD. Varicella zoster virus (VZV) infects and establishes latency in enteric neurons. J Neurovirol **2011**; 17:578-89.

13. Weinberg A, Levin MJ. VZV T cell-mediated immunity. In: Abendroth A, Arvin AM, Moffat JF, eds. Current topics in microbiology and immunology. 2010/05/18 ed. Berlin, Heidelberg: Springer-Verlag, **2010**:341-57.

14. Head H, Campbell AW. The pathology of herpes zoster and its bearing on sensory localisation. Brain **1900**; 23:353-523.

15. Hope-Simpson RE. The nature of herpes zoster: a long-term study and a new hypothesis. Proc R Soc Med **1965**; 58:9-20.

16. Denny-Brown D, Adams RD, Fitzgerald PJ. Pathologic features of herpes zoster: a note on "geniculate herpes". Arch Neuro Psychiatr **1944**; 51:216-31.

17. Gnann JW, Jr., Whitley RJ. Clinical practice. Herpes zoster. N Engl J Med **2002**; 347:340-6.

18. Oxman MN. Zoster vaccine: current status and future prospects. Clin Infect Dis **2010**; 51:197-213.

19. Luby JP, Ramierez-Ronda C, Rinner S. A longitudinal study of varicella-zoster virus infections in renal transplant recipients. J Infect Dis **1977**; 135:659-63.

20. Gershon AA, Steinberg SP, Borkowsky W, Lennette D, Lennette E. IgM to varicella-zoster virus: demonstration in patients with and without clinical zoster. Pediatr Infect Dis **1982**; 1:164-7.

21. Ljungman P, Lonnquist B, Gahrton G. Clinical and subclinical reactivations of varicella-zoster virus in immunocompromised patients. J Infect Dis **1986**; 153:840-7.

22. Hayward A, Levin M, Wolf W, Angelova G, Gilden D. Varicella-zoster virus-specific immunity after herpes zoster. J Infect Dis **1991**; 163:873-5.

23. Wilson A, Sharp M, Koropchak CM. Subclinical varicella-zoster virus viremia, herpes zoster, and T-lymphocyte immunity to varicella-zoster viral antigens after bone marrow transplantation. J Infect Dis **1992**; 165:119-26.

24. Cohrs RJ, Mehta SK, Schmid DS, Gilden DH, Pierson DL. Asymptomatic reactivation and shed of infectious varicella zoster virus in astronauts. J Med Virol **2008**; 80:1116-22.

25. Gershon AA, Gershon MD, Shapiro E. Live attenuated varicella vaccine: prevention of varicella and of zoster. Current Publication **2021**.

26. Good RA, Zak SJ. Disturbances in gamma globulin synthesis as experiments of nature. Pediatrics **1956**; 18:109-49.

27. Ochs HD, Smith CI. X-linked agammaglobulinemia. A clinical and molecular analysis. Medicine (Baltimore) **1996**; 75:287-99.

28. Burke BL, Steele RW, Beard OW, Wood JS, Cain TD, Marmer DJ. Immune responses to varicella-zoster in the aged. Arch Intern Med **1982**; 142:291-3.

29. Vossen MT, Biezeveld MH, de Jong MD, et al. Absence of circulating natural killer and primed CD8+ cells in life-threatening varicella. J Infect Dis **2005**; 191:198-206.

30. Laing KJ, Ouwendijk WJD, Koelle DM, Verjans G. Immunobiology of varicella-zoster virus infection. J Infect Dis **2018**; 218:S68-S74.

31. Levin MJ. Zoster vaccines. In: Plotkin S, Orenstein W, Offit P, Edwards KM, eds. Plotkin's vaccines. 7th ed. Philadelphia, PA: Elsevier, **2018**:1268-81.e6.

32. Arvin AM, Koropchak CM, Williams BR, Grumet FC, Foung SK. Early immune response in healthy and immunocompromised subjects with primary varicella-zoster virus infection. J Infect Dis **1986**; 154:422-9.

33. Weinberg A, Zhang JH, Oxman MN, et al. Varicella-zoster virus-specific immune responses to herpes zoster in elderly participants in a trial of a clinically effective zoster vaccine. J Infect Dis **2009**; 200:1068-77.

34. Oxman MN. Clinical manifestations of herpes zoster. In: Arvin AM, Gershon AA, eds. Varicella-zoster virus: virology and clinical management. Cambridge, UK: Cambridge University Press, **2000**:246-75.

35. Arvin AM. Cell-mediated immunity to varicella-zoster virus. J Infect Dis **1992**; 166:S35-S41.

36. Levin MJ. Varicella-zoster virus and virus DNA in the blood and oropharynx of people with latent or active varicella-zoster virus infections. J Clin Virol **2014**; 61:487-95.

37. Gershon AA, LaRussa P, Steinberg S, Mervish N, Lo SH, Meier P. The protective effect of immunologic boosting against zoster: an analysis in leukemic children who were vaccinated against chickenpox. J Infect Dis **1996**; 173:450-3.

38. Gershon AA, Steinberg SP, Gelb L. Clinical reinfection with varicella-zoster virus. J Infect Dis **1984**; 149:137-42.

39. Weigle KA, Grose C. Molecular dissection of the humoral immune response to individual varicella-zoster viral proteins during chickenpox, quiescence, reinfection, and reactivation. J Infect Dis **1984**; 149:741-9.

40. Terada K, Kawano S, Yoshihiro K, Morita T. Proliferative response to varicella-zoster virus is inverse related to development of high levels of varicella-zoster virus specific IgG antibodies. Scand J Infect Dis **1993**; 25:775-8.

41. Terada K, Niizuma T, Yagi Y, Miyashima H, Kataoka N, Sadahiro T. Low induction of varicella-zoster virus-specific secretory IgA antibody after vaccination. J Med Virol **2000**; 62:46-51.

42. Thomas SL, Wheeler JG, Hall AJ. Contacts with varicella or with children and protection against herpes zoster in adults: a case-control study. Lancet **2002**; 360:678-82.

43. Vossen MT, Gent MR, Weel JF, de Jong MD, van Lier RA, Kuijpers TW. Development of virus-specific CD4+ T cells on reexposure to Varicella-Zoster virus. J Infect Dis **2004**; 190:72-82.

44. Ogunjimi B, Smits E, Hens N, et al. Exploring the impact of exposure to primary varicella in children on varicella-zoster virus immunity of parents. Viral Immunol **2011**; 24:151-7.

45. Ogunjimi B, Smits E, Heynderickx S, et al. Influence of frequent infectious exposures on general and varicella-zoster virus-specific immune responses in pediatricians. Clin Vaccine Immunol **2014**; 21:417-26.

46. Ogunjimi B, Van den Bergh J, Meysman P, et al. Multidisciplinary study of the secondary immune response in grandparents re-exposed to chickenpox. Sci Rep **2017**; 7:1077.

47. Elias G, Souquette A, Heynderickx S, et al. Altered CD4(+) T cell immunity in nurses occupationally exposed to viral pathogens. Clin Exp Immunol **2018**; 194:192-204.

48. Brisson M, Gay NJ, Edmunds WJ, Andrews NJ. Exposure to varicella boosts immunity to herpes-zoster: implications for mass vaccination against chickenpox. Vaccine **2002**; 20:2500-7.

49. Harpaz R. Do varicella vaccination programs change the epidemiology of herpes zoster? A comprehensive review, with focus on the United States. Expert Rev Vaccines **2019**; 18:793-811.

50. Ragozzino MW, Melton LJ, 3rd, Kurland LT, Chu CP, Perry HO. Population-based study of herpes zoster and its sequelae. Medicine **1982**; 61:310-6.

51. Jumaan AO, Yu O, Jackson LA, Bohlke K, Galil K, Seward JF. Incidence of herpes zoster, before and after varicella-vaccination-associated decreases in the incidence of varicella, 1992-2002. J Infect Dis **2005**; 191:2002-7.

52. Leung J, Harpaz R, Molinari NA, Jumaan A, Zhou F. Herpes zoster incidence among insured persons in the United States, 1993-2006: evaluation of impact of varicella vaccination. Clin Infect Dis **2011**; 52:332-40.

53. Hales CM, Harpaz R, Joesoef MR, Bialek SR. Examination of links between herpes zoster incidence and childhood varicella vaccination. Ann Intern Med **2013**; 159:739-45.

54. Kawai K, Yawn BP, Wollan P, Harpaz R. Increasing incidence of herpes zoster over a 60-year period from a population-based study. Clin Infect Dis **2016**; 63:221-6.

55. Harpaz R, Leung JW. The epidemiology of herpes zoster in the United States during the era of varicella and herpes zoster vaccines: changing patterns among older adults. Clin Infect Dis **2019**; 69:341-4.

56. Wolfson LJ, Daniels VJ, Altland A, Black W, Huang W, Ou W. The impact of varicella vaccination on the incidence of varicella and herpes zoster in the United States: updated evidence from observational databases, 1991-2016. Clin Infect Dis **2020**; 70:995-1002.

57. Thompson RR, Kong CL, Porco TC, Kim E, Ebert CD, Acharya NR. Herpes zoster and post-herpetic neuralgia: changing incidence rates from 1994 to 2018 in the United States. Clin Infect Dis **2020**.

58. Kawai K, Gebremeskel BG, Acosta CJ. Systematic review of incidence and complications of herpes zoster: towards a global perspective. BMJ Open **2014**; 4:e004833.

59. Brisson M, Edmunds WJ, Law B, et al. Epidemiology of varicella zoster virus infection in Canada and the United Kingdom. Epidemiol Infect **2001**; 127:305-14.

60. Chao DY, Chien YZ, Yeh YP, Hsu PS, Lian IB. The incidence of varicella and herpes zoster in Taiwan during a period of increasing varicella vaccine coverage, 2000-2008. Epidemiol Infect **2012**; 140:1131-40.

61. Gaillat J, Gajdos V Fau - Launay O, Launay O Fau - Malvy D, et al. Does monastic life predispose to the risk of Saint Anthony's fire (herpes zoster)? Clin Infect Dis **2011**; 53:405-10.

62. van Oorschot D, Vroling H, Bunge E, Diaz-Decaro J, Curran D, Yawn B. A systematic literature review of herpes zoster incidence worldwide. Hum Vaccin Immunother **2021**; 17:1714-32.

63. Oxman MN, Williams HM, Levin MJ, et al. Efficacy of zoster vaccine according to dermatome region. 45th Interscience Conference of Antimicrobial Agents and Chemotherapy. Washington, DC, **2005**.

64. Harpaz R, Ortega-Sanchez IR, Seward JF. Prevention of herpes zoster: recommendations of the Advisory Committee on Immunization Practices (ACIP). MMWR Recomm Rep **2008**; 57:1-30; quiz CE2-4.

65. Centers for Disease Control and Prevention. Herpes zoster Updated January 2020. In: Hamborsky J, Kroger A, Wolfe C, eds. Epidemiology and prevention of vaccine-preventable diseases (The Pink Book). 13th ed, **2015**.

66. Yawn BP, Gilden D. The global epidemiology of herpes zoster. Neurology **2013**; 81:928-30.

67. Thomas SL, Hall AJ. What does epidemiology tell us about risk factors for herpes zoster? Lancet Infect Dis **2004**; 4:26-33.

68. Gershon AA, Gershon MD, Breuer J, Levin MJ, Oaklander AL, Griffiths PD. Advances in the understanding of the pathogenesis and epidemiology of herpes zoster. J Clin Virol **2010**; 48 Suppl 1:S2-S7.

69. Haanpaa M, Nurmikko T, Hurme M. Polymorphism of the IL-10 gene is associated with susceptibility to herpes zoster. Scand J Infect Dis **2002**; 34:112-4.

70. Weinberg A, Lazar AA, Zerbe GO, et al. Influence of age and nature of primary infection on varicella-zoster virus-specific cell-mediated immune responses. J Infect Dis **2010**; 201:1024-30.

71. Goronzy JJ, Weyand CM. Understanding immunosenescence to improve responses to vaccines. Nat Immunol **2013**; 14:428-36.

72. Cho JW, Shin DH, Lee KS. Polymorphism of the IL-10 gene is associated with susceptibility to herpes zoster in Korea. J Dermatol Sci **2007**; 45:213-5.

73. Hicks LD, Cook-Norris RH, Mendoza N, Madkan V, Arora A, Tyring SK. Family history as a risk factor for herpes zoster: a case-control study. Arch Dermatol **2008**; 144:603-8.

74. Hernandez PO, Javed S, Mendoza N, Lapolla W, Hicks LD, Tyring SK. Family history and herpes zoster risk in the era of shingles vaccination. J Clin Virol **2011**; 52:344-8.

75. Rubicz R, Leach CT, Kraig E, et al. Genetic factors influence serological measures of common infections. Hum Hered **2011**; 72:133-41.

76. Crosslin DR, Carrell DS, Burt A, et al. Genetic variation in the HLA region is associated with susceptibility to herpes zoster. Genes Immun **2015**; 16:1-7.

77. Meysman P, De Neuter N, Bartholomeus E, et al. Increased herpes zoster risk associated with poor HLA-A immediate early 62 protein (IE62) affinity. Immunogenetics **2018**; 70:363-72.

78. Harpaz R, Leung JW. The epidemiology of herpes zoster in the United States during the Era of varicella and herpes zoster vaccines: changing patterns among children. Clin Infect Dis **2019**; 69:345-7.

79. Drolet M, Brisson M, Schmader KE, et al. The impact of herpes zoster and postherpetic neuralgia on health-related quality of life: a prospective study. CMAJ **2010**; 182:1731-6.

80. Hope-Simpson RE. Postherpetic neuralgia. J R Coll Gen Pract **1975**; 25:571-5.

81. Kost RG, Straus SE. Postherpetic neuralgia--pathogenesis, treatment, and prevention. N Engl J Med **1996**; 335:32-42.

82. Cohen JI. Clinical practice: herpes zoster. N Engl J Med **2013**; 369:255-63.

83. Johnson RW, Rice AS. Clinical practice. Postherpetic neuralgia. N Engl J Med **2014**; 371:1526-33.

84. Forbes HJ, Thomas SL, Smeeth L, et al. A systematic review and meta-analysis of risk factors for postherpetic neuralgia. Pain **2016**; 157:30-54.

85. Dworkin RH, Portenoy RK. Pain and its persistence in herpes zoster. Pain **1996**; 67:241-51.

86. Johnson RW, Levin MJ. Herpes zoster and its prevention by vaccination. Interdiscip Top Gerontol Geriatr **2020**; 43:131-45.

87. Harvey M, Prosser LA, Rose AM, Ortega-Sanchez IR, Harpaz R. Aggregate health and economic burden of herpes zoster in the United States: illustrative example of a pain condition. Pain **2020**; 161:361-8.

88. Watson CP, Oaklander AL. Postherpetic neuralgia. Pain Pract **2002**; 2:295-307.

89. Schmader KE, Sloane R, Pieper C, et al. The impact of acute herpes zoster pain and discomfort on functional status and quality of life in older adults. Clin J Pain **2007**; 23:490-6.

90. Dooling KL, Guo A, Patel M, et al. Recommendations of the Advisory Committee on Immunization Practices for Use of Herpes Zoster Vaccines. MMWR Morb Mortal Wkly Rep **2018**; 67:103-8.

91. Oxman MN, Levin MJ, Johnson GR, et al. A vaccine to prevent herpes zoster and postherpetic neuralgia in older adults. N Engl J Med **2005**; 352:2271-84.

92. Takahashi M, Otsuka T, Okuno Y, Asano Y, Yazaki T. Live vaccine used to prevent the spread of varicella in children in hospital. Lancet **1974**; 2:1288-90.

93. Takahashi M, Okuno Y, Otsuka T, Osame J, Takamizawa A. Development of a live attenuated varicella vaccine. Biken J **1975**; 18:25-33.

94. Takahashi M, Asano Y, Kamiya H, et al. Development of varicella vaccine. J Infect Dis **2008**; 197 Suppl 2:41-4.

95. Berger R, Luescher D, Just M. Enhancement of varicella-zoster-specific immune responses in the elderly by boosting with varicella vaccine. J Infect Dis **1984**; 149:647-.

96. Weibel RE, Neff BJ, Kuter BJ, et al. Live attenuated varicella virus vaccine. Efficacy trial in healthy children. N Engl J Med **1984**; 310:1409-15.

97. Asano Y, Nagai T, Miyata T, et al. Long-term protective immunity of recipients of the OKA strain of live varicella vaccine. Pediatrics **1985**; 75:667-71.

98. Arbeter AM, Starr SE, Plotkin SA. Varicella vaccine studies in healthy children and adults. Pediatrics **1986**; 78:748-56.

99. Takahashi M. Clinical overview of varicella vaccine: development and early studies. Pediatrics **1986**; 78:736-41.

100. Gershon AA, Steinberg SP, LaRussa P, Ferrara A, Hammerschlag M, Gelb L. Immunization of healthy adults with live attenuated varicella vaccine. J Infect Dis **1988**; 158:132-7.

101. Levin MJ, Murray M, Rotbart HA, Zerbe GO, White CJ, Hayward AR. Immune response of elderly individuals to a live attenuated varicella vaccine. J Infect Dis **1992**; 166:253-9.

102. Takahashi M, Iketani T, Sasada K, et al. Immunization of the elderly and patients with collagen vascular diseases with live varicella vaccine and use of varicella skin antigen. J Infect Dis **1992**; 166 Suppl 1:58-62.

103. Levin MJ, Barber D, Goldblatt E, et al. Use of a live attenuated varicella vaccine to boost varicella-specific immune responses in seropositive people 55 years of age and older: duration of booster effect. J Infect Dis **1998**; 178 Suppl 1:109-12.

104. Trannoy E, Berger R, Hollander G, et al. Vaccination of immunocompetent elderly subjects with a live attenuated Oka strain of varicella zoster virus: a randomized, controlled, dose-response trial. Vaccine **2000**; 18:1700-6.

105. Takahashi M, Okada S, Miyagawa H, et al. Enhancement of immunity against VZV by giving live varicella vaccine to the elderly assessed by VZV skin test and IAHA, gpELISA antibody assay. Vaccine **2003**; 21:3845-53.

106. Levin MJ, Gershon AA, Weinberg A, Song L-Y, Fentin T, Nowak B. Administration of live varicella vaccine to HIV-infected children with current or past significant depression of CD4(+) T cells. J Infect Dis **2006**; 194:247-55.

107. Gershon AA, Levin MJ, Weinberg A, et al. A phase I-II study of live attenuated varicella-zoster virus vaccine to boost immunity in human immunodeficiency virus-infected children with previous varicella. The Pediatric infectious disease journal **2009**; 28:653-5.

108. Marin M, Guris D, Chaves SS, et al. Prevention of varicella: recommendations of the Advisory Committee on Immunization Practices (ACIP). MMWR Recomm Rep **2007**; 56:1-40.

109. Leung J, Bialek SR, Marin M. Trends in varicella mortality in the United States: Data from vital statistics and the national surveillance system. Hum Vaccin Immunother **2015**; 11:662-8.

110. Shapiro ED, Vazquez M, Esposito D, et al. Effectiveness of 2 doses of varicella vaccine in children. J Infect Dis **2011**; 203:312-5.

111. Gershon AA. Is chickenpox so bad, what do we know about immunity to varicella zoster virus, and what does it tell us about the future? J Infect **2017**; 74 Suppl 1:S27-S33.

112. Coplan PM, Schmader K, Nikas A, et al. Development of a measure of the burden of pain due to herpes zoster and postherpetic neuralgia for prevention trials: adaptation of the brief pain inventory. J Pain **2004**; 5:344-56.

113. Schmader KE, Johnson GR, Saddier P, et al. Effect of a zoster vaccine on herpes zoster-related interference with functional status and health-related quality-of-life measures in older adults. J Am Geriatr Soc **2010**; 58:1634-41.

114. Simberkoff MS, Arbeit RD, Johnson GR, et al. Safety of herpes zoster vaccine in the shingles prevention study: a randomized trial. Ann Intern Med **2010**; 152:545-54.

115. Morrison VA, Oxman MN, Levin MJ, et al. Safety of zoster vaccine in elderly adults following documented herpes zoster. J Infect Dis **2013**; 208:559-63.

116. Mills R, Tyring SK, Levin MJ, et al. Safety, tolerability, and immunogenicity of zoster vaccine in subjects with a history of herpes zoster. Vaccine **2010**; 28:4204-9.

117. Schmader KE, Levin MJ, Gnann JW, et al. Efficacy, safety, and tolerability of herpes zoster vaccine in persons aged 50-59 years. Clin Infect Dis **2012**; 54:922-8.

118. Levin MJ, Oxman MN, Zhang JH, et al. Varicella-zoster virus-specific immune responses in elderly recipients of a herpes zoster vaccine. J Infect Dis **2008**; 197:825-35.

119. Levin MJ. Immune senescence and vaccines to prevent herpes zoster in older persons. Curr Opin Immunol **2012**; 24:494-500.

120. Hales CM, Harpaz R, Ortega-Sanchez I, Bialek SR. Update on recommendations for use of herpes zoster vaccine. MMWR Morb Mortal Wkly Rep **2014**; 63:729-31.

121. Freedman MS, Ault K, Bernstein H. Advisory Committee on Immunization Practices Recommended Immunization Schedule for Adults Aged 19 Years or Older - United States, 2021. MMWR Morb Mortal Wkly Rep **2021**; 70:193-6.

122. Schmader KE, Oxman MN, Levin MJ, et al. Persistence of the efficacy of zoster vaccine in the shingles prevention study and the short-term persistence substudy. Clin Infect Dis **2012**; 55:1320-8.

123. Morrison VA, Johnson GR, Schmader KE, et al. Long-term persistence of zoster vaccine efficacy. Clin Infect Dis **2015**; 60:900-9.

124. Tseng HF, Smith N, Harpaz R, Bialek SR, Sy LS, Jacobsen SJ. Herpes zoster vaccine in older adults and the risk of subsequent herpes zoster disease. JAMA **2011**; 305:160-6.

125. Langan SM, Smeeth L, Margolis DJ, Thomas SL. Herpes zoster vaccine effectiveness against incident herpes zoster and post-herpetic neuralgia in an older US population: a cohort study. PLoS Med **2013**; 10:e1001420.

126. Marin M, Yawn BP, Hales CM, et al. Herpes zoster vaccine effectiveness and manifestations of herpes zoster and associated pain by vaccination status. Hum Vaccin Immunother **2015**; 11:1157-64.

127. Tseng HF, Lewin B, Hales CM, et al. Zoster vaccine and the risk of postherpetic neuralgia in patients who developed herpes zoster despite having received the zoster vaccine. J Infect Dis **2015**; 212:1222-31.

128. Tseng HF, Harpaz R, Luo Y, et al. Declining effectiveness of herpes zoster vaccine in adults aged ≥60 years. J Infect Dis **2016**; 213:1872-5.

129. Izurieta HS, Wernecke M, Kelman J, et al. Effectiveness and duration of protection provided by the live-attenuated herpes zoster vaccine in the Medicare population ages 65 years and older. Clin Infect Dis **2017**; 64:785-93.

130. Baxter R, Bartlett J, Fireman B, et al. Long-Term Effectiveness of the Live Zoster Vaccine in Preventing Shingles: A Cohort Study. Am J Epidemiol **2018**; 187:161-9.

131. Klein NP, Bartlett J, Fireman B, et al. Long-term effectiveness of zoster vaccine live for postherpetic neuralgia prevention. Vaccine **2019**; 37:5422-7.

132. Andrews N, Stowe J, Kuyumdzhieva G, et al. Impact of the herpes zoster vaccination programme on hospitalised and general practice consulted herpes zoster in the 5 years after its introduction in England: a population-based study. BMJ Open **2020**; 10:e037458.

133. Arvin AM, Oliver S, Reichelt M, et al. Analysis of the functions of glycoproteins E and I and their promoters during VZV replication in vitro and in skin and T-cell xenografts in the SCID mouse model of VZV pathogenesis. In: Abendroth A, Arvin AM, Moffat JF, eds. Current Topics in Microbiology and Immunology. Berlin Heidelberg: Springer-Verlag, **2010**:129-46.

134. Leroux-Roels I, Leroux-Roels G, Clement F, et al. A phase 1/2 clinical trial evaluating safety and immunogenicity of a varicella zoster glycoprotein e subunit vaccine candidate in young and older adults. J Infect Dis **2012**; 206:1280-90.

135. Didierlaurent AM, Collignon C, Bourguignon P, et al. Enhancement of adaptive immunity by the human vaccine adjuvant AS01 depends on activated dendritic cells. J Immunol **2014**; 193:1920-30.

136. Vandepapelière P, Horsmans Y, Moris P, et al. Vaccine adjuvant systems containing monophosphoryl lipid A and QS21 induce strong and persistent humoral and T cell responses against hepatitis B surface antigen in healthy adult volunteers. Vaccine **2008**; 26:1375-86.

137. Lal H, Cunningham AL, Godeaux O, et al. Efficacy of an adjuvanted herpes zoster subunit vaccine in older adults. N Engl J Med **2015**; 372:2087-96.

138. Cunningham AL, Lal H, Kovac M, et al. Efficacy of the herpes zoster subunit vaccine in adults 70 years of age or older. N Engl J Med **2016**; 375:1019-32.

139. Cunningham AL, Heineman T. Vaccine profile of herpes zoster (HZ/su) subunit vaccine. Expert Rev Vaccines **2017**; 16:1-10.

140. Lopez-Fauqued M, Campora L, Delannois F, et al. Safety profile of the adjuvanted recombinant zoster vaccine: Pooled analysis of two large randomised phase 3 trials. Vaccine **2019**; 37:2482-93.

141. Godeaux O, Kovac M, Shu D, et al. Immunogenicity and safety of an adjuvanted herpes zoster subunit candidate vaccine in adults ≥ 50 years of age with a prior history of herpes zoster: a phase III, non-randomized, open-label clinical trial. Hum Vaccin Immunother **2017**; 13:1051-8.

142. Izurieta HS, Wu X, Forshee R, et al. Recombinant zoster vaccine (Shingrix) real-world effectiveness in the first two years post-licensure. Clin Infect Dis, **2021**:ciab125.

143. Sun Y, Kim E, Kong CL, Arnold BF, Porco TC, Acharya NR. Effectiveness of the recombinant zoster vaccine in adults aged 50 and older in the United States: a claims-based cohort study. Clin Infect Dis **2021**.

144. Chlibek R, Bayas JM, Collins H, et al. Safety and immunogenicity of an AS01-adjuvanted varicella-zoster virus subunit candidate vaccine against herpes zoster in adults >=50 years of age. J Infect Dis **2013**; 208:1953-61.

145. Lal H, Zahaf T, Heineman TC. Safety and immunogenicity of an AS01-adjuvanted varicella zoster virus subunit candidate vaccine (HZ/su): a phase-I, open-label study in Japanese adults. Hum Vaccin Immunother **2013**; 9:1425-9.

146. Chlibek R, Smetana J, Pauksens K, et al. Safety and immunogenicity of three different formulations of an adjuvanted varicella-zoster virus subunit candidate vaccine in older adults: a phase II, randomized, controlled study. Vaccine **2014**; 32:1745-53.

147. Weinberg A, Kroehl ME, Johnson MJ, et al. Comparative immune responses to licensed herpes zoster vaccines. J Infect Dis **2018**; 218:S81-S7.

148. Schmid DS, Miao C, Leung J, Johnson M, Weinberg A, Levin MJ. Comparative antibody responses to the live-attenuated and recombinant herpes zoster vaccines. J Virol **2021**; 95:e00240-21.

149. Chlibek R, Pauksens K, Rombo L, et al. Long-term immunogenicity and safety of an investigational herpes zoster subunit vaccine in older adults. Vaccine **2016**; 34:863-8.

150. Schwarz TF, Volpe S, Catteau G, et al. Persistence of immune response to an adjuvanted varicella-zoster virus subunit vaccine for up to year nine in older adults. Hum Vaccin Immunother **2018**; 14:1370-7.

151. Bastidas A, Catteau G, Volpe S, et al. Long-term immunological persistence of the adjuvanted recombinant zoster vaccine: clinical data and mathematical modeling. Open Forum Infect Dis **2019**; 6 Suppl 2:S84–S5.

152. Hastie A, Catteau G, Enemuo A, et al. Immunogenicity of the adjuvanted recombinant zoster vaccine: persistence and anamnestic response to additional doses administered 10 years after primary vaccination. J Infect Dis, **2020**:jiaa300.

153. Levin MJ, Schmader KE, Pang L, et al. Cellular and humoral responses to a second dose of herpes zoster vaccine administered 10 years after the first dose among older adults. J Infect Dis **2016**; 213:14-22.

154. Weinberg A, Popmihajlov Z, Schmader KE, et al. Persistence of varicella-zoster virus cell-mediated immunity after the administration of a second dose of live herpes zoster vaccine. J Infect Dis **2019**; 219:335-8.

155. Grupping K, Campora L, Douha M, et al. Immunogenicity and safety of the HZ/su adjuvanted herpes zoster subunit vaccine in adults previously vaccinated with a live attenuated herpes zoster vaccine. J Infect Dis **2017**; 216:1343-51.

156. Dagnew AF, Klein NP, Herve C, et al. The adjuvanted recombinant zoster vaccine in adults aged ≥65 years previously vaccinated with a live-attenuated herpes zoster vaccine. J Infect Dis, **2020**:jiaa083.

157. Chen SY, Suaya JA, Li Q, et al. Incidence of herpes zoster in patients with altered immune function. Infection **2014**; 42:325-34.

158. Levin MJ, Bresnitz E, Popmihajlov Z, et al. Studies with herpes zoster vaccines in immune compromised patients. Expert Rev Vaccines **2017**; 16:1217-30.

159. Cheetham TC, Marcy SM, Tseng HF, et al. Risk of herpes zoster and disseminated varicella zoster in patients taking immunosuppressant drugs at the time of zoster vaccination. Mayo Clin Proc **2015**; 90:865-73.

160. Ljungman P, Cordonnier C, Einsele H, et al. Vaccination of hematopoietic cell transplant recipients. Bone Marrow Transplant **2009**; 44:521-6.

161. Zhang J, Delzell E, Xie F, et al. The use, safety, and effectiveness of herpes zoster vaccination in individuals with inflammatory and autoimmune diseases: a longitudinal observational study. Arthritis Res Ther **2011**; 13:R174.

162. Naidus E, Damon L, Schwartz BS, Breed C, Liu C. Experience with use of Zostavax((R)) in patients with hematologic malignancy and hematopoietic cell transplant recipients. Am J Hematol **2012**; 87:123-5.

163. Zhang J, Xie F, Delzell E, et al. Association between vaccination for herpes zoster and risk of herpes zoster infection among older patients with selected immune-mediated diseases. JAMA **2012**; 308:43-9.

164. Issa NC, Marty FM, Leblebjian H, et al. Live attenuated varicella-zoster vaccine in hematopoietic stem cell transplantation recipients. Biol Blood Marrow Transplant **2014**; 20:285-7.

165. Perry LM, Winthrop KL, Curtis JR. Vaccinations for rheumatoid arthritis. Curr Rheumatol Rep **2014**; 16:431.

166. Shafran SD. Live attenuated herpes zoster vaccine for HIV-infected adults. HIV Med **2016**; 17:305-10.

167. Tsigrelis C, Ljungman P. Vaccinations in patients with hematological malignancies. Blood Rev **2016**; 30:139-47.

168. Grint DJ, McDonald HI, Walker JL, Amirthalingam G, Andrews N, Thomas S. Safety of inadvertent administration of live zoster vaccine to immunosuppressed individuals in a UK-based observational cohort analysis. BMJ Open **2020**; 10:e034886.

169. Miller G, Schaefer H, Yoder S, et al. A randomized, placebo-controlled phase I trial of live, attenuated herpes zoster vaccine in subjects with end-stage renal disease immunized prior to renal transplantation. Transpl Infect Dis **2018**; 20:e12874.

170. Tseng HF, Luo Y, Shi J, et al. Effectiveness of herpes zoster vaccine in patients 60 years and older with end-stage renal disease. Clin Infect Dis **2016**; 62:462-7.

171. Redman RL, Nader S, Zerboni L, et al. Early reconstitution of immunity and decreased severity of herpes zoster in bone marrow transplant recipients immunized with inactivated varicella vaccine. J Infect Dis **1997**; 176:578-85.

172. Hata A, Asanuma H, Rinki M, et al. Use of an inactivated varicella vaccine in recipients of hematopoietic-cell transplants. New Engl J Med **2002**; 347:26-34.

173. Mullane KM, Winston DJ, Wertheim MS, et al. Safety and immunogenicity of heat-treated zoster vaccine (ZVHT) in immunocompromised adults. J Infect Dis **2013**; 208:1375-85.

174. Parrino J, McNeil SA, Lawrence SJ, et al. Safety and immunogenicity of inactivated varicella-zoster virus vaccine in adults with hematologic malignancies receiving treatment with anti-CD20 monoclonal antibodies. Vaccine **2017**; 35:1764-9.

175. Eberhardson M, Hall S, Papp KA, et al. Safety and immunogenicity of inactivated varicella-zoster virus vaccine in adults with autoimmune disease: a phase 2, randomized, double-blind, placebo-controlled clinical trial. Clin Infect Dis **2017**; 65:1174-82.

176. Winston DJ, Mullane KM, Cornely OA, et al. Inactivated varicella zoster vaccine in autologous haemopoietic stem-cell transplant recipients: an international, multicentre, randomised, double-blind, placebo-controlled trial. Lancet **2018**; 391:2116-27.

177. Mullane KM, Morrison VA, Camacho LH, et al. Safety and efficacy of inactivated varicella zoster virus vaccine in immunocompromised patients with malignancies: a two-arm, randomised, double-blind, phase 3 trial. Lancet Infect Dis **2019**; 19:1001-12.

178. Boeckh MJ, Arvin AM, Mullane KM, et al. Immunogenicity of inactivated varicella zoster vaccine in autologous hematopoietic stem cell transplant recipients and patients with solid or hematologic cancer. Open Forum Infect Dis **2020**; 7:ofaa172.

179. Stadtmauer EA, Sullivan KM, Marty FM, et al. A phase 1/2 study of an adjuvanted varicella-zoster virus subunit vaccine in autologous hematopoietic cell transplant recipients. Blood **2014**; 124:2921-9.

180. Berkowitz EM, Moyle G, Stellbrink HJ, et al. Safety and immunogenicity of an adjuvanted herpes zoster subunit candidate vaccine in HIV-infected adults: a phase 1/2a randomized, placebo-controlled study. J Infect Dis **2015**; 211:1279-87.

181. Bastidas A, de la Serna J, El Idrissi M, et al. Effect of recombinant zoster vaccine on incidence of herpes zoster after autologous stem cell transplantation: a randomized clinical trial. JAMA **2019**; 322:123-33.

182. Dagnew AF, Ilhan O, Lee WS, et al. Immunogenicity and safety of the adjuvanted recombinant zoster vaccine in adults with haematological malignancies: a phase 3, randomised, clinical trial and post-hoc efficacy analysis. Lancet Infect Dis **2019**; 19:988-1000.

183. Vink P, Delgado Mingorance I, Maximiano Alonso C, et al. Immunogenicity and safety of the adjuvanted recombinant zoster vaccine in patients with solid tumors, vaccinated before or during chemotherapy: a randomized trial. Cancer **2019**; 125:1301-12.

184. Vink P, Ramon Torrell JM, Sanchez Fructuoso A, et al. Immunogenicity and safety of the adjuvanted recombinant zoster vaccine in chronically immunosuppressed adults following renal transplant: a phase 3, randomized clinical trial. Clin Infect Dis **2020**; 70:181-90.

185. Curran D, Matthews S, Rowley SD, et al. Recombinant zoster vaccine significantly reduces the impact on quality of life caused by herpes zoster in adult autologous hematopoietic stem cell transplant recipients: a randomized placebo-controlled trial (ZOE-HSCT). Biol Blood Marrow Transplant **2019**; 25:2474-81.

186. Baumrin E, Izaguirre NE, Bausk B, et al. Safety and reactogenicity of the recombinant zoster vaccine after allogeneic hematopoietic cell transplantation. Blood Adv **2021**; 5:1585-93.

187. Gebo KA, Kalyani R, Moore RD, Polydefkis MJ. The incidence of, risk factors for, and sequelae of herpes zoster among HIV patients in the highly active antiretroviral therapy era. J Acquir Immune Defic Syndr **2005**; 40:169-74.

188. Centers for Disease Control and Prevention. HIV infection and adult vaccination. Available at: https://www.cdc.gov/vaccines/adults/rec-vac/health-conditions/hiv.html. Accessed 24 Mar 2021.

189. Rubin LG, Levin MJ, Ljungman P, et al. 2013 IDSA clinical practice guideline for vaccination of the immunocompromised host. Clin Infect Dis **2014**; 58:309-18.

190. Geretti AM, Brook G, Cameron C, et al. British HIV Association guidelines on the use of vaccines in HIV-positive adults 2015. HIV Med **2016**; 17:S2-S81.

191. Thompson MA, Horberg MA, Agwu AL, et al. Primary care guidance for persons with human immunodeficiency virus: 2020 update by the HIV Medicine Association of the Infectious Diseases Society of America. Clin Infect Dis, **2020**.

192. Weinberg A, Levin MJ, Macgregor RR. Safety and immunogenicity of a live attenuated varicella vaccine in VZV-seropositive HIV-infected adults. Hum Vaccin **2010**; 6:318-21.

193. Benson CA, Andersen JW, Macatangay BJC, et al. Safety and immunogenicity of zoster vaccine live in human immunodeficiency virus-infected adults with CD4+ cell counts >200 cells/ml virologically suppressed on antiretroviral therapy. Clin Infect Dis **2018**; 67:1712-9.

194. Aziz M, Kessler H, Huhn G. Providers' lack of knowledge about herpes zoster in HIV-infected patients is among barriers to herpes zoster vaccination. Int J STD AIDS **2013**; 24:433-9.

195. Erlandson KM, Streifel A, Novin AR, et al. Low rates of vaccination for herpes zoster in older people living with HIV. AIDS Res Hum Retroviruses **2018**; 34:603-6.

196. Hawkins KL, Gordon KS, Levin MJ, et al. Herpes zoster and herpes zoster vaccine rates among adults living with and without HIV in the Veterans Aging Cohort Study. J Acquir Immune Defic Syndr **2018**; 79:527-33.

197. Strangfeld A, Listing J, Herzer P, et al. Risk of herpes zoster in patients with rheumatoid arthritis treated with anti-TNF-alpha agents. JAMA **2009**; 301:737-44.

198. Curtis JR, Xie F, Yun H, Bernatsky S, Winthrop KL. Real-world comparative risks of herpes virus infections in tofacitinib and biologic-treated patients with rheumatoid arthritis. Ann Rheum Dis **2016**; 75:1843-7.

199. Yun H, Yang S, Chen L, et al. Risk of herpes zoster in auto-immune and inflammatory diseases: implications for vaccination. Arthritis Rheumatol **2016**; 68:2328-37.

200. Calabrese LH, Abud-Mendoza C, Lindsey SM, et al. Live zoster vaccine in patients with rheumatoid arthritis treated with tofacitinib with or without methotrexate, or adalimumab with methotrexate: a post hoc analysis of data from a phase IIIb/IV randomized study. Arthritis Care Res (Hoboken) **2020**; 72:353-9.

201. Winthrop KL, Wouters AG, Choy EH, et al. The safety and immunogenicity of live zoster vaccination in patients with rheumatoid arthritis before starting tofacitinib: a randomized phase II trial. Arthritis Rheumatol **2017**; 69:1969-77.

202. Winthrop KL, Wouters A, Choy EH, et al. Long-term effectiveness of live herpes zoster vaccine in patients with rheumatoid arthritis subsequently treated with tofacitinib. Ann Rheum Dis **2020**; 79:669-71.

203. Curtis J, Bridges SL, Cofield SS, et al. Results from a randomized controlled trial of the safety of live varicella vaccine in TNF-treated patients [abstract]. Arthritis Rheumatol **2019**; 71 (Suppl 10).

204. Guthridge JM, Cogman A, Merrill JT, et al. Herpes zoster vaccination in SLE: a pilot study of immunogenicity. J Rheumatol **2013**; 40:1875-80.

205. Mok CC, Chan KH, Ho LY, Fung YF, Fung WF, Woo PCY. Safety and immune response of a live-attenuated herpes zoster vaccine in patients with systemic lupus erythematosus: a randomised placebo-controlled trial. Ann Rheum Dis **2019**; 78:1663-8.

206. Israeli E, Agmon-Levin N, Blank M, Shoenfeld Y. Adjuvants and autoimmunity. Lupus **2009**; 18:1217-25.

207. Dagnew AF, Rausch D, Herve C, et al. Efficacy and serious adverse events profile of the adjuvanted recombinant zoster vaccine in adults with pre-existing potential immune-mediated diseases: a pooled post hoc analysis on two parallel randomized trials. Rheumatology (Oxford) **2021**; 60:1226-33.

208. Stevens E, Weinblatt ME, Massarotti E, Griffin F, Emani S, Desai S. Safety of the zoster vaccine recombinant adjuvanted in rheumatoid arthritis and other systemic rheumatic disease patients: a single center's experience with 400 patients. ACR Open Rheumatol **2020**; 2:357-61.

209. Lenfant T, Jin Y, Kirchner E, Hajj-Ali RA, Calabrese LH, Calabrese C. Safety of recombinant zoster vaccine: a retrospective study of 622 rheumatology patients. Rheumatology (Oxford): Oxford University Press, **2021**:keab139.

210. Satyam VR, Li PH, Reich J, et al. Safety of recombinant zoster vaccine in patients with inflammatory bowel disease. Dig Dis Sci **2020**; 65:2986-91.

211. GlaxoSmithKline. Briefing document. Shingrix* (zoster vaccine recombinant, aduvanted) vaccines and related biological products Advisory Committee. Meeting Date: September 13, 2017.

212. Didierlaurent AM, Desssart C, Cunningham AL. Clarification regarding the statement of the association between the recombinant zoster vaccine (RZV) and gout flares. Ann Rheum Dis, **2019**:annrheumdis-2019-216639.

213. Yokose C, McCormick N, Chen C, et al. Risk of gout flares after vaccination: a prospective case cross-over study. Ann Rheum Dis **2019**; 78:1601-4.

214. Willis ED, Woodward M, Brown E, et al. Herpes zoster vaccine live: a 10 year review of post-marketing safety experience. Vaccine **2017**; 35:7231-9.

215. Totterdell J, Phillips A, Glover C, et al. Safety of live attenuated herpes zoster vaccine in adults 70-79 years: a self-controlled case series analysis using primary care data from Australia's MedicineInsight program. Vaccine **2020**; 38:3968-79.

216. Tseng HF, Schmid DS, Harpaz R, et al. Herpes zoster caused by vaccine-strain varicella zoster virus in an immunocompetent recipient of zoster vaccine. Clin Infect Dis **2014**; 58:1125-8.

217. Ortiz-Brizuela E, Leal-Vega F, Cuellar-Rodríguez J, Bobadilla-Del-Valle M, Ponce-de-León A. Vaccine-derived varicella zoster infection in a kidney transplant recipient after zoster vaccine live administration. Vaccine **2019**; 37:3576-9.

218. Costa E, Buxton J, Brown J, Templeton KE, Breuer J, Johannessen I. Fatal disseminated varicella zoster infection following zoster vaccination in an immunocompromised patient. BMJ Case Rep **2016**:bcr2015212688.

219. Alexander KE, Tong PL, Macartney K, Beresford R, Sheppeard V, Gupta M. Live zoster vaccination in an immunocompromised patient leading to death secondary to disseminated varicella zoster virus infection. Vaccine **2018**; 36:3890-3.

220. Dubey V, MacFadden D. Disseminated varicella zoster virus infection after vaccination with a live attenuated vaccine. CMAJ **2019**; 191:E1025-E7.

221. Hesse EM, Shimabukuro TT, Su JR, et al. Postlicensure safety surveillance of recombinant zoster vaccine (Shingrix) - United States, October 2017-June 2018. MMWR Morb Mortal Wkly Rep **2019**; 68:91-4.

222. Lehmann A, Matoba A. Reactivation of herpes zoster stromal keratitis after HZ/su adjuvanted herpes zoster subunit vaccine. Ophthalmology **2018**; 125:1682.

223. Heydari-Kamjani M, Vante I, Uppal P, Demory Beckler M, Kesselman MM. Uveitis sarcoidosis presumably initiated after administration of Shingrix vaccine. Cureus **2019**; 11:e4920.

224. Bell H, Kamal N, Wong U. Blistering autoimmune skin reaction following SHINGRIX vaccination in an ulcerative colitis patient: case report and literature review. Vaccine **2020**; 38:7455-7.

225. Chen RI, Deaner JD, Srivastava SK, Lowder CY. Acute retinal necrosis following recombinant subunit varicella-zoster virus vaccine. Am J Ophthalmol Case Rep **2020**; 20:100962.

226. Housel LA, McClenathan BM. Herpes zoster after recombinant zoster vaccine: a first case report. J Allergy Clin Immunol Pract **2020**; 8:772-4.e1.

227. Kohn D, Wetzig T. [Zoster disease after Shingrix vaccination]. Hautarzt **2020**.

228. Riano I, Cristancho C, Treadwell T. Stevens-Johnson Syndrome-like reaction after exposure to pembrolizumab and recombinant zoster vaccine in a patient with metastatic lung cancer. J Investig Med High Impact Case Rep. Vol. 8, **2020**:1-4.

229. Rajaratnam N, Govil S, Patel R, Ahmed M, Elias S. Rhabdomyolysis after recombinant zoster vaccination: a rare adverse reaction. J Community Hosp Intern Med Perspect **2021**; 11:145-6.

230. U.S. Food and Drug Administration. FDA requires a warning about Guillain-Barré Syndrome (GBS) be Included in the prescribing information for Shingrix®. Available at: https://www.fda.gov/vaccines-blood-biologics/safety-availability-biologics/fda-requires-warning-about-guillain-barre-syndrome-gbs-be-included-prescribing-information-shingrix. Accessed 25 Mar 2021.

231. GlaxoSmithKline. Shingrix® package insert. Available at: https://www.fda.gov/media/108597/download. Accessed 25 Mar 2021.

232. Kroger AT, Atkinson WL, Marcuse EK, Pickering LK. General recommendations on immunization: recommendations of the Advisory Committee on Immunization Practices (ACIP). MMWR Recomm Rep **2006**; 55:1-48.

233. MacIntyre CR, Egerton T, McCaughey M, et al. Concomitant administration of zoster and pneumococcal vaccines in adults ≥60 years old. Hum Vaccin **2010**; 6:894-902.

234. Oxman MN, Gershon AA, Poland GA. Zoster vaccine recommendations: the importance of using a clinically valid correlate of protection. Vaccine **2011**; 29:3625-7.

235. Tseng HF, Smith N, Sy LS, Jacobsen SJ. Evaluation of the incidence of herpes zoster after concomitant administration of zoster vaccine and polysaccharide pneumococcal vaccine. Vaccine **2011**; 29:3628-32.

236. Bruxvoort K, Sy LS, Luo Y, Tseng HF. Real-world evidence for regulatory decisions: concomitant administration of zoster vaccine live and pneumococcal polysaccharide vaccine. Am J Epidemiol **2018**; 187:1856-62.

237. Merck & Co. I. Zostavax® package insert. Available at: https://www.merck.com/product/usa/pi_circulars/z/zostavax/zostavax_pi2.pdf. Accessed 25 Mar-2021.

238. Kerzner B, Murray AV, Cheng E, et al. Safety and immunogenicity profile of the concomitant administration of ZOSTAVAX and inactivated influenza vaccine in adults aged 50 and older. J Am Geriatr Soc **2007**; 55:1499-507.

239. Levin MJ, Buchwald UK, Gardner J, et al. Immunogenicity and safety of zoster vaccine live administered with quadrivalent influenza virus vaccine. Vaccine **2018**; 36:179-85.

240. Maréchal C, Lal H, Poder A, et al. Immunogenicity and safety of the adjuvanted recombinant zoster vaccine co-administered with the 23-valent pneumococcal polysaccharide vaccine in adults ≥50 years of age: A randomized trial. Vaccine **2018**; 36:4278-86.

241. Strezova A, Lal H, Enweonye I, et al. The adjuvanted recombinant zoster vaccine co-administered with a tetanus, diphtheria and pertussis vaccine in adults aged >/=50 years: a randomized trial. Vaccine **2019**; 37:5877-85.

242. Schmitt HJ, Schuind A, Knuf M, et al. Clinical experience of a tricomponent acellular pertussis vaccine combined with diphtheria and tetanus toxoids for primary vaccination in 22,505 infants. J Pediatr **1996**; 129:695-701.

243. Schwarz TF, Aggarwal N, Moeckesch B, et al. Immunogenicity and safety of an adjuvanted herpes zoster subunit vaccine coadministered with seasonal influenza vaccine in adults aged 50 years or older. J Infect Dis **2017**; 216:1352-61.

244. Centers for Disease Control and Prevention. Administering Shingrix. Available at: https://www.cdc.gov/vaccines/vpd/shingles/hcp/shingrix/administering-vaccine.html. Accessed 21 Jun 2021.

245. Doherty TM, Connolly MP, Del Giudice G, et al. Vaccination programs for older adults in an era of demographic change. Eur Geriatr Med **2018**; 9:289-300.

246. Cassimos DC, Effraimidou E, Medic S, Konstantinidis T, Theodoridou M, Maltezou HC. Vaccination programs for adults in Europe, 2019. Vaccines (Basel) **2020**; 8:34.

247. European Centre for Disease Prevention and Control. Herpes zoster: recommended vaccinations. Available at: https://vaccine-schedule.ecdc.europa.eu/Scheduler/ByDisease?SelectedDiseaseId=51&SelectedCountryIdByDisease=-1. Accessed 25 Mar 2021.

248. Bundesministerium Soziales Gesundheit Pflege und Konsumentenschutz (BMSGPK). Impfplan Österreich 2021, **2021**.

249. Superior Health Council. Vaccination against herpes zoster virus (zona). Available at: https://www.health.belgium.be/sites/default/files/uploads/fields/fpshealth_theme_file/9209_shc_advice_9209_zonaa5_pdt.pdf.

250. Terviseamet. What can adults be vaccinated against? Vaccine-preventable diseases. Available at: https://ta.vaktsineeri.ee/en/vaccination-adults/what-can-adults-be-vaccinated-against. Accessed 25 Mar 2021.

251. Martins D, McCormack D, Tadrous M, et al. Impact of a publicly funded herpes zoster immunization program on the burden of disease in Ontario, Canada: a population-based study. Clin Infect Dis **2021**; 72:279-84.

252. National Institute of Infectious Diseases. Varicella/herpes zoster: epidemiological trends and vaccines. Infectious Agents Surveillance Report. Vol. 39, **2018**:129-30.

253. Watanabe D, Mizukami A, Holl K, et al. The potential public health impact of herpes zoster vaccination of people aged >/= 50 years in Japan: results of a Markov model analysis. Dermatol Ther (Heidelb) **2018**; 8:269-84.

254. Sadaoka T, Mori Y. Vaccine development for varicella-zoster virus. Adv Exp Med Biol **2018**; 1045:123-42.

255. Choi WS, Choi JH, Jung DS, et al. Immunogenicity and safety of a new live attenuated herpes zoster vaccine (NBP608) compared to Zostavax(R) in healthy adults aged 50 years and older. Vaccine **2019**; 37:3605-10.

256. Warrington R, Ismail S, on behalf of the National Advisory Committee on Immunization. Summary of the NACI update on herpes zoster vaccines. Can Commun Dis Rep **2018**; 44:220-5.

257. Ständige Impfkommission: Empfehlungen der Ständigen Impfkommission (STIKO) am Robert Koch-Institut. Epid Bull **2019**; 34:313-64.

258. Polack FP, Thomas SJ, Kitchin N, et al. Safety and efficacy of the BNT162b2 mRNA Covid-19 vaccine. N Engl J Med **2020**; 383:2603-15.

259. Baden LR, El Sahly HM, Essink B, et al. Efficacy and safety of the mRNA-1273 SARS-CoV-2 vaccine. N Engl J Med **2021**; 384:403-16.

260. Monslow MA, Elbashir S, Sullivan NL, et al. Immunogenicity generated by mRNA vaccine encoding VZV gE antigen is comparable to adjuvanted subunit vaccine and better than live attenuated vaccine in nonhuman primates. Vaccine **2020**; 38:5793-802.

261. Lee SJ, Park HJ, Ko HL, et al. Evaluation of glycoprotein E subunit and live attenuated varicella-zoster virus vaccines formulated with a single-strand RNA-based adjuvant. Immun Inflamm Dis **2020**; 8:216-27.

262. Privor-Dumm LA, Poland GA, Barratt J, et al. A global agenda for older adult immunization in the COVID-19 era: a roadmap for action. Vaccine, **2020**:S0264-410X(20)30885-9.

263. United Nations Department of Economic and Social Affairs Population Division (ST/ESA/SER.A/423). World population prospects 2019: highlights. New York: United Nations, **2019**:iii, 39.

264. Yawn BP, Saddier P, Wollan PC, St Sauver JL, Kurland MJ, Sy LS. A population-based study of the incidence and complication rates of herpes zoster before zoster vaccine introduction. Mayo Clin Proc **2007**; 82:1341-9.

265. Katz J, Cooper EM, Walther RR, Sweeney EW, Dworkin RH. Acute pain in herpes zoster and its impact on health-related quality of life. Clin Infect Dis **2004**; 39:342-8.

266. Schmader K, Gnann JW, Jr., Watson CP. The epidemiological, clinical, and pathological rationale for the herpes zoster vaccine. J Infect Dis **2008**; 197 Suppl 2:S207-S15.

267. United Nations Department of Economic and Social Affairs Population Division. World population prospects 2019. Available at: https://population.un.org/wpp/Download/Probabilistic/Population/. Accessed 15 Jun 2021.

268. Prosser LA, Harpaz R, Rose AM, et al. A cost-effectiveness analysis of vaccination for prevention of herpes zoster and related complications: input for national recommendations. Ann Intern Med **2019**; 170:380-8.

269. Chiyaka ET, Nghiem VT, Zhang L, Deshpande A, Mullen PD, Le P. Cost-effectiveness of herpes zoster vaccination: a systematic review. Pharmacoeconomics **2019**; 37:169-200.

270. World Health Organization. Regional strategic plan for immunization 2014 - 2020. Available at: http://www.afro.who.int/sites/default/files/2017-06/oms-ivb-rvap-afro-en-20150408_final_sent140317_0.pdf?ua=1. Accessed 25 Mar 2021.

271. Tsolia M, Gershon AA, Steinberg SP, Gelb L. Live attenuated varicella vaccine: evidence that the virus is attenuated and the importance of skin lesions in transmission of varicella-zoster virus. National Institute of Allergy and Infectious Diseases Varicella Vaccine Collaborative Study Group. J Pediatr **1990**; 116:184-9.

272. Seward JF, Zhang JX, Maupin TJ, Mascola L, Jumaan AO. Contagiousness of varicella in vaccinated cases: a household contact study. JAMA **2004**; 292:704-8.

273. Marin M, Leung J, Gershon AA. Transmission of vaccine-strain varicella-zoster virus: a systematic review. Pediatrics **2019**; 144:e20191305.

274. Weinmann S, Chun C, Schmid DS, et al. Incidence and clinical characteristics of herpes zoster among children in the varicella vaccine era, 2005-2009. J Infect Dis **2013**; 208:1859-68.

275. Weinmann S, Naleway AL, Koppolu P, et al. Incidence of herpes zoster among children: 2003-2014. Pediatrics **2019**; 144:e20182917.

276. Artavanis-Tsakonas K, Tongren JE, Riley EM. The war between the malaria parasite and the immune system: immunity, immunoregulation and immunopathology. Clin Exp Immunol **2003**; 133:145-52.

277. Gowda DC, Wu X. Parasite recognition and signaling mechanisms in innate immune responses to malaria. Front Immunol **2018**; 9:3006.

278. Brace PT, Tezera LB, Bielecka MK, et al. Mycobacterium tuberculosis subverts negative regulatory pathways in human macrophages to drive immunopathology. PLoS Pathog **2017**; 13:e1006367.

279. Chao WC, Yen CL, Wu CH, Shieh CC. How mycobacteria take advantage of the weakness in human immune system in the modern world. J Microbiol Immunol Infect **2020**; 53:209-15.

280. Migueles SA, Connors M. Success and failure of the cellular immune response against HIV-1. Nat Immunol **2015**; 16:563-70.

281. Rogan DC, Connors M. Immunologic control of HIV-1: what have we learned and can we induce It? Curr HIV/AIDS Rep, **2021**.

282. Zheng YQ, Naguib YW, Dong Y, Shi YC, Bou S, Cui Z. Applications of bacillus Calmette-Guerin and recombinant bacillus Calmette-Guerin in vaccine development and tumor immunotherapy. Expert Rev Vaccines **2015**; 14:1255-75.

283. Mustafa AS. BCG as a vector for novel recombinant vaccines against infectious diseases and cancers. Vaccines (Basel) **2020**; 8:1-5.

284. Nguyen TT, Oh Y, Kim Y, Shin Y, Baek SK, Park JH. Progress in microneedle array patch (MAP) for vaccine delivery. Hum Vaccin Immunother **2021**; 17:316-27.
