# Supplementary material for: Herpes Zoster Vaccines
Source: J Infect Dis. 2021 Sep 30;224(Suppl 4):S429–42. doi: 10.1093/infdis/jiab387 (PMC8482024; doi:10.1093/infdis/jiab387)
Supplement: jiab387_suppl_Supplementary-Table-1 [file jiab387_suppl_supplementary-table-1.docx]

**SUPPLEMENTARY TABLE 1.** **Clinical Trials Assessing Zoster Vaccines in Immunocompromised Patients**

| **Trial Number** | **Vaccine** | **Study Design** | **Intervention** | **Participants** | **N** | **Age** | **Outcome Measures** | **Outcomes** | **Reference** |
| --- | --- | --- | --- | --- | --- | --- | --- | --- | --- |
| N/N | VVHT (VARIVAX, 2900 pfu/0.5 mL; heat-inactivated) | Randomized trial | single dose of VVHT at 1 M after BMT;  or 3 doses of VVHT at 1, 2, 3 M after BMT | patients with auto-BMT/PBSC or allo-BMT | single dose: 28 (VVHT:14; unvaccinated:14); 3 doses: 47 (VVHT:24; unvaccinated: 23) | 18-49 yoa | incidence of HZ; severity of HZ | single dose: no difference in incidence or severity of HZ between vaccinated and unvaccinated; immunogenic;  no SAE;  3 doses: no difference in incidence of HZ; significant reduction in HZ severity; immunogenic;  no SAE | Redman RL, Nader S, Zerboni L, et al. J Infect Dis 1997; 176:578-85 |
| N/N | VVHT (6115 pfu/0.5 mL; heat-inactivated) | Randomized trial | 4 doses of VVHT: dose 1 within30 D prior to HSCT; doses 2-4 at 30, 60, and 90 D post-transplant | auto-HSCT recipients | 119  (VVHT:59; unvaccinated:60) | 18-60 yoa | incidence of HZ within 12M post-transplant | Immunogenic (higher VZV-CMI in vaccine compared to placebo recipients); reduction in the incidence of HZ | Hata A, Asanuma H, Rinki M, et al. New Engl J Med 2002; 347:26-34 |
| NCT00535236 | ZVHT | Phase I, randomized, double-blind, placebo-controlled | 4 doses ZVHT or placebo ~30 D apart; for HSCT recipients, dose 1 ~30 D prior to HSCT and doses 2-4 at 30, 60, and 90 D post-HSCT | adults with either: STM; HM; HIV with CD4+ T-cell counts of ≤200; auto-HSCT or allo-HSCT | 341  [ZVHT:262 (STM:59; HM:62;HIV:60;  auto-HSCT:40; allo-HSCT:41); placebo: 79 (STM:20; HM:19;HIV:20; auto-HSCT:10; allo-HSCT:10)] | ≥18 yoa | safety, immunogenicity | generally safe and immunogenic through 28 days post-dose 4 in adults with STM, HM, and HIV; auto-HSCT but not allo-HSCT patients showed rise in T-cell response; no increase in antibody responses in either HSCT population | Mullane KM, Winston DJ, Wertheim MS, et al. J Infect Dis 2013; 208:1375-85 |

| **Trial Number** | **Vaccine** | **Study Design** | **Intervention** | **Participants** | **N** | **Age** | **Outcome Measures** | **Outcomes** | **Reference** |
| --- | --- | --- | --- | --- | --- | --- | --- | --- | --- |
| NCT01460719 | ZVIN | Phase I,  open-label, single-arm | 4 doses of ZVIN; dose 1 at ~5 D prior to any chemotherapy dose in the cycle. Doses 2 through 4 administered ~ every 30 D and ~5 D prior to upcoming dose of chemotherapy | patients with HM receiving treatment with anti-CD20 mono-clonal antibodies | 80 | ≥18 yoa | safety, immunogenicity | well-tolerated; elicited statistically significant VZV-specific T-cell responses 28D post-dose 4 | Parrino J, McNeil SA, Lawrence SJ, et al. Vaccine 2017; 35:1764-9 |
| NCT01527383 | ZVIN  or  ZVIN high antigen (Ag) | Phase II, randomized, double-blind, placebo-controlled | 4 doses of ZVIN or ZVIN high Ag dose  or placebo | patients with AI disease on AI treatment (≥1 biologic agent, with or without nonbiologic immunosuppressive therapy or ≥1 nonbiologic with no biologic agents) | 354  (ZVIN:190;  ZVIN high Ag:102; placebo:62) | ≥18 yoa | safety, immunogenicity | well tolerated and immunogenic; VZV-specific immune responses generally comparable between patients receiving biologic and nonbiologic therapy;  ZVIN high Ag excluded from immunogenicity analysis | Eberhardson M, Hall S, Papp KA, et al. Clin Infect Dis 2017; 65:1174-82 |
| NCT00851786 | ZVL | Phase II, randomized, double-blind, placebo-controlled | 2 doses of ZVL, 6 weeks apart | patients with HIV infection with CD4+ T-cell counts of ≥200 cells/μL  on ART | 395  (ZVL:296;  placebo:99) | ≥18 yoa | safety; immunogenicity | safe and immunogenic;  VZV antibody (gpELISA) titers after ZVL dose 2 comparable to those after dose 1 | Benson CA, Andersen JW, Macatangay BJC, et al. Clin Infect Dis 2018; 67:1712-9 |
| NCT01229267 | ZVIN  or  ZVIN high antigen (Ag) | Phase III, randomized, double-blind, placebo-controlled | 4 doses of ZVIN or ZVIN high Ag  or placebo (5:1:5); first dose 5-60 D pre-transplant; doses 2-4 at 30, 60, 90 D post-transplant | auto-HSCT recipients | 1230  (ZVIN:560;  ZVIN high Ag:106; placebo:564) | ≥18 yoa | efficacy,  safety | VE for HZ incidence: 63.8%;  VE for HZ-related complications: 73.5%;  VE for PHN: 83.7%;  no safety concerns | Winston DJ, Mullane KM, Cornely OA, et al. Lancet 2018; 391:2116-27; Boeckh MJ, Arvin AM, Mullane KM, et al. Open Forum Infect Dis 2020; 7:ofaa172 |
| **Trial Number** | **Vaccine** | **Study Design** | **Intervention** | **Participants** | **N** | **Age** | **Outcome Measures** | **Outcomes** | **Reference** |
| NCT01254630 | ZVIN | Phase III, two-arm, randomized, double-blind, placebo-controlled | 4 doses of ZVIN ~30 D apart; or dose 1 at ~5 D prior to any chemotherapy dose in the cycle. Doses 2 through 4 were administered ~ every 20-40 D and ~5 D prior to upcoming dose of chemotherapy | patients with STM or HM | STM: 2712 (ZVIN:1348; placebo:1364);  HM: 2573 (ZVIN:1288; placebo:1285) | ≥18 yoa | efficacy for incidence of HZ; safety; immunogenicity | STM: VE = 63.3%; immunogenic; no safety concerns; HM: safe and immunogenic but trial was terminated after interim analysis (no reduction in HZ incidence) | Mullane KM, Morrison VA, Camacho LH, et al. Lancet Infect Dis 2019; 19:1001-12; Boeckh MJ, Arvin AM, Mullane KM, et al. Open Forum Infect Dis 2020; 7:ofaa172 |
| DMID 09-0016 | ZVL | Phase I, randomized, double-blinded,  placebo-controlled | 1 dose of ZVL ≥4 weeks prior to renal transplant | patients with chronic kidney disease awaiting transplant | 40  (ZVL: 32;  placebo: 8) | ≥18 yoa | safety, immunogenicity | safe and well tolerated; immunogenic, based on anti-  VZV antibody responses | Miller G, Schaefer H, Yoder S, et al. Transpl Infect Dis 2018; 20:e12874 |
| NCT02477150 | ZVL | Phase IV,  randomized, double-blinded,  placebo-controlled | 1 dose of ZVL or placebo | patients with SLE with stable immunosuppressive treatment for ≥6 M | 90  (ZVL: 45;  placebo: 45) | ≥18 yoa | safety, immunogenicity | safe and well tolerated; immunogenic; no increase in flares after vaccination | Mok CC, Chan KH, Ho LY, et al. Ann Rheum Dis 2019; 78:1663-8 |
| NCT00920218 | RZV  (gE/AS01B or gE/AS01E) | Phase I/IIa, randomized, observer-blind, placebo-controlled | 3 doses of gE/AS01B or gE/AS01E or  1 dose saline and 2 doses gE/AS01B  or placebo  at 2M, 3M and 5M post-transplant | auto-HSCT recipients | 120  (gE/AS01B 3-dose:30;  gE/AS01E 3-dose:29; gE/AS01B:31 2-dose:31;  placebo:30 | ≥18 yoa | safety, immunogenicity | Both formulations, both schedules well tolerated and immunogenic;  gE/AS01B more immunogenic than gE/AS01E;  less immunogenic in patients with non-Hodgkin B-cell lymphoma | Stadtmauer EA, Sullivan KM, Marty FM, et al. Blood 2014; 124:2921-9 |
| NCT01165203 | RZV | Phase I/IIa, randomized 3:2, observer-blind, placebo-controlled | 3 doses of RZV or  3 doses placebo | patients with HIV infection | 123  [RZV:74 (ART/high CD4: 56;  ART/low CD4: 9;  ART-/high CD4: 9);  Placebo: 49 (ART/high CD4: 38;  ART/low CD4: 5;  ART-/high CD4: 6)] | ≥18 yoa | safety, immunogenicity | Immunogenic; acceptable safety profile; immune responses persist over prevaccination levels ≥1 year after dose 3. | Berkowitz EM, Moyle G, Stellbrink HJ, et al. J Infect Dis 2015; 211:1279-87 |

| **Trial Number** | **Vaccine** | **Study Design** | **Intervention** | **Participants** | **N** | **Age** | **Outcome Measures** | **Outcomes** | **Reference** |
| --- | --- | --- | --- | --- | --- | --- | --- | --- | --- |
| NCT01610414 | RZV | Phase III, randomized, observer-blinded, placebo-controlled | 2 doses of RZV or placebo; 1–2 M apart; first dose 50-70 D posttransplant | auto-HSCT recipients | 1846  (RZV:922; placebo:924) | ≥18 yoa | efficacy, immunogenicity, safety^a)^;  QoL^b)^; | ^a)^VE: 68.2%; immunogenic; generally well tolerated;  ^b)^QoL: shorter duration and less severity of HZ; less interference with ADL; higher QoL scores in RZV vs. placebo recipients with HZ | ^a)^Bastidas A, de la Serna J, El Idrissi M, et al. JAMA 2019; 322:123-33;  ^b)^Curran D, Matthews S, Rowley SD, et al. Biol Blood Marrow Transplant 2019; 25:2474-81. |
| NCT01767467 | RZV | Phase III, randomized, observer-blind placebo-controlled | 2 doses of RZV or placebo 1–2 M apart | patients with HM | 562 [RZV:286 (102 during and 181 after IS treatment); placebo:279 (106 during and 173 after treatment)] | ≥18 yoa | safety, reactogenicity, immunogenicity | immunogenic;  more reactogenic than placebo; acceptable safety profile;  posthoc analysis: VE = 87.2% (but small numbers) | Dagnew AF, Ilhan O, Lee WS, et al. Lancet Infect Dis 2019; 19:988-1000 |
| NCT01798056 | RZV | Phase II/III, randomized, observer-blind, placebo-controlled | 2 doses of RZV  or placebo;  first dose 8-30 D prior to start of chemotherapy cycle (RZV-PreChemo, Placebo-PreChemo); or first dose within  1 D of start of chemotherapy cycle (RZV-OnChemo, Placebo-OnChemo) | patients with STM | 262  (RZV-PreChemo:103, RZV-OnChemo:27; placebo-PreChemo:108; placebo-OnChemo:24) | ≥18 yoa | safety, immunogenicity | immunogenic through 12M post-vaccination; no safety concerns | Vink P, Delgado Mingorance I, Maximiano Alonso C, et al. Cancer 2019; 125:1301-12 |
| NCT02058589 | RZV | Phase III, randomized, observer-blind, placebo-controlled | 2 doses of RZV or placebo 1–2 M apart, 4–18 M post-transplant | renal transplant recipients | 264 (RZV:132; placebo: 132) | ≥18 yoa | safety, immunogenicity | immunogenic through 12 M post-vaccination; no safety concerns | Vink P, Ramon Torrell JM, Sanchez Fructuoso A, et al. Clin Infect Dis 2020; 70:181-90. |

**ADL**: activities of daily living; **AI**: auto-immune; **allo-HSCT**: allogeneic hematopoietic stem-cell transplant; **ART**: antiretroviral therapy; **auto-HSCT**: autologous hematopoietic stem-cell transplant; **BMT**: bone marrow transplant; **D**: day(s); **gE**: glycoprotein E; **gpELISA**: VZV glycoprotein enzyme-linked immunosorbent antibody;  **HIV**: human immunodeficiency virus; **HM**: hematologic malignancy; **HSCT**: hematopoietic stem-cell transplant; **HZ**: herpes zoster; **M**: month(s); **N/N**: no trial number; **PBSC**: peripheral blood stem cell infusion; **QoL**: quality of life; **RZV**: recombinant adjuvanted zoster vaccine; **SAE**: serious adverse event(s); **SLE:** systemic lupus erythematosus**; STM**: solid tumor malignancy; **VE**: vaccine efficacy; **VVHT**: heat-treated varicella vaccine; **VZV**; varicella-zoster virus; **VZV-CMI**: VZV-specific cell mediated immunity; **yoa**: years of age; **ZVHT**: heat-treated zoster vaccine; **ZVIN**: zoster vaccine inactivated by γ irradiation; **ZVL**: live attenuated zoster vaccine.
