# Supplementary material for: Herpes Zoster Vaccines
Source: J Infect Dis. 2021 Sep 30;224(Suppl 4):S429–42. doi: 10.1093/infdis/jiab387 (PMC8482024; doi:10.1093/infdis/jiab387)
Supplement: jiab387_suppl_Supplementary-Table-2 [file jiab387_suppl_supplementary-table-2.docx]

**SUPPLEMENTARY TABLE 2. Countries in which ZVL (Zostavax®) and RZV (Shingrix®) are Licensed and Distributed**

| **COUNTRY** | **% of Population ≥65 yoa^a)^** | **Country Aging Status^b)^** | **ZOSTER VACCINE** | | | |
| --- | --- | --- | --- | --- | --- | --- |
|  |  |  | **ZVL (Zostavax®)** | | **RZV (Shingrix®)** | |
|  |  |  | **Licensed** | **Sold** | **Licensed** | **Administered** |
| Argentina | 11.2 | Aging | ✓ | ✓ |  |  |
| Australia | 15.9 | Aged | ✓ | ✓ | ✓ |  |
| Austria | 19.1 | Aged | ✓ | ✓ | ✓ |  |
| Belgium | 19.0 | Aged | ✓ | ✓ | ✓ | ✓ |
| Bermuda | 16.8* | Aged |  | ✓ |  |  |
| Brazil | 9.3 | Aging | ✓ | ✓ |  |  |
| Brunei | 5.2 | N/A | ✓ |  |  |  |
| Bulgaria | 21.3 | Super-Aged | ✓ |  | ✓ |  |
| Canada | 17.6 | Aged | ✓ | ✓ | ✓ | ✓ |
| Chile | 11.9 | Aging | ✓ | ✓ |  |  |
| China | 11.5 | Aging |  |  | ✓ | ✓ |
| Colombia | 8.8 | Aging | ✓ | ✓ |  |  |
| Croatia | 20.9 | Aged | ✓ |  | ✓ |  |
| Cyprus | 14.0 | Aging | ✓ |  | ✓ |  |
| Czech Republic | 19.8 | Aged | ✓ |  | ✓ |  |
| Denmark | 20.0 | Aged | ✓ | ✓ | ✓ |  |
| Estonia | 20.0 | Aged | ✓ | ✓ | ✓ |  |
| Finland | 22.1 | Super-Aged | ✓ | ✓ | ✓ |  |
| France | 20.4 | Aged | ✓ | ✓ | ✓ |  |
| Germany | 21.6 | Super-Aged | ✓ | ✓ | ✓ | ✓ |
| Greece | 21.9 | Super-Aged | ✓ | ✓ | ✓ |  |
| Hong Kong | 17.5 | Aged | ✓ | ✓ | ✓ |  |
| Hungary | 19.7 | Aged | ✓ |  | ✓ |  |
| Iceland | 15.2 | Aged | ✓ |  |  |  |
| India | 6.4 | N/A | ✓ |  |  |  |
| Ireland | 14.2 | Aged | ✓ | ✓ | ✓ |  |
| Israel | 12.2 | Aging | ✓ | ✓ |  |  |
| Italy | 23.0 | Super-Aged | ✓ | ✓ | ✓ |  |
| Japan | 28.0 | Super-Aged |  |  | ✓ | ✓ |
| Korea, Republic of | 15.1 | Aged | ✓ | ✓ |  |  |
| Kuwait | 2.8 | N/A | ✓ |  |  |  |
| Latvia | 20.3 | Aged | ✓ |  | ✓ |  |
| Liechtenstein | 18.9** | Aged | ✓ |  |  |  |
| Lithuania | 20.2 | Aged | ✓ | ✓ | ✓ |  |
| Luxembourg | 14.3 | Aged | ✓ |  | ✓ |  |
| Malaysia | 6.9 | N/A | ✓ | ✓ |  |  |
| Malta | 20.8 | Aged | ✓ |  | ✓ |  |
| Mexico | 7.4 | Aging | ✓ | ✓ |  |  |
| Netherlands | 19.6 | Aged | ✓ | ✓ | ✓ | ✓ |
| New Zealand | 16.0 | Aged | ✓ | ✓ | ✓ |  |
| Norway | 17.3 | Aged | ✓ | ✓ |  |  |
| **COUNTRY** | **% of Population ≥65 YOA^a)^** | **Country Aging Status^b)^** | **ZOSTER VACCINE** | | | |
|  |  |  | **ZVL (Zostavax®)** | | **RZV (Shingrix®)** | |
|  |  |  | **Licensed** | **Sold** | **Licensed** | **Administered** |
| Oman | 2.4 | N/A | ✓ |  |  |  |
| Philippines | 5.3 | N/A | ✓ | ✓ |  |  |
| Poland | 18.1 | Aged | ✓ |  | ✓ |  |
| Portugal | 22.4 | Super-Aged | ✓ | ✓ | ✓ |  |
| Puerto Rico | 19.7 | Aged |  | ✓ |  |  |
| Qatar | 1.5 | N/A | ✓ |  |  |  |
| Romania | 18.8 | Aged | ✓ |  | ✓ |  |
| Singapore | 12.4 | Aging | ✓ | ✓ | ✓ |  |
| Slovakia | 16.2 | Aged | ✓ |  | ✓ |  |
| Slovenia | 20.2 | Aged | ✓ | ✓ | ✓ |  |
| South Africa | 5.4 | N/A | ✓ | ✓ |  |  |
| Spain | 19.6 | Aged | ✓ | ✓ | ✓ |  |
| Sweden | 20.2 | Aged | ✓ | ✓ | ✓ | ✓ |
| Switzerland | 18.8 | Aged | ✓ | ✓ |  |  |
| Taiwan | 15.3*** | Aged | ✓ | ✓ |  |  |
| Thailand | 12.4 | Aging | ✓ | ✓ |  |  |
| Turkey | 8.7 | Aging | ✓ |  |  |  |
| United Arab Emirates | 1.2 | N/A | ✓ |  |  |  |
| United Kingdom | 18.5 | Aged |  | ✓ |  |  |
| United States | 16.2 | Aged | ✓ |  | ✓ | ✓ |
| Venezuela | 7.6 | Aging | ✓ |  |  |  |

^a)^World Bank Data: Populations 65 and Above https://data.worldbank.org/indicator/SP.POP.65UP.TO.ZS, accessed 1Mar 2021

^b)^WHO's convention for categorizing countries by aging (based on percent of adults ≥65 years of age (yoa) in the total population): Aging: >7%-14%; Aged: >14%-21%; Super-Aged: >21%

* Based on the 2016 Bermuda Census https://www.gov.bm/bermuda-census, accessed 11 Mar 2021

** https://www.indexmundi.com/liechtenstein/demographics_profile.html, accessed 22 Feb 2021

*** Statistical Yearbook of the Republic of China https://eng.stat.gov.tw/lp.asp?CtNode=6340&CtUnit=1072&BaseDSD=36&mp=5, accessed 22 Feb 2021
